# A sex/gender perspective on interventions to promote children’s and adolescents’ overall physical activity: results from genEffects systematic review

**DOI:** 10.1186/s12887-020-02370-9

**Published:** 2020-10-10

**Authors:** Carolin Schulze, Yolanda Demetriou, Sandra Emmerling, Annegret Schlund, Susan P. Phillips, Lorri Puil, Stephanie E. Coen, Anne K. Reimers

**Affiliations:** 1 Institute of Human Movement Science and Health, Chemnitz University of Technology, Thüringer Weg 11, D-09126 Chemnitz, Germany; 2 Department of Sport and Health Sciences, Technical University of Munich, Munich, Germany; 3 Centre for Studies in Primary Care, School of Medicine, Queen’s University, Kingston, Canada; 4 Department of Anesthesiology, Pharmacology & Therapeutics, Faculty of Medicine, University of British Columbia, Vancouver, Canada; 5 School of Geography, University of Nottingham/UK, Nottingham, UK; 6 Department of Sport Science and Sport, Friedrich-Alexander-University Erlangen-Nuremberg, Erlangen, Germany

**Keywords:** Sport, Exercise, Boys, Girls, Health, Equity, Intervention, Physical activity

## Abstract

**Background:**

To evaluate the effects of interventions on children’s and adolescents’ overall physical activity (PA) for boys and girls separately and to appraise the extent to which the studies haven taken sex/gender into account.

**Methods:**

Systematic review and semi-quantitative analysis. Eleven electronic databases were searched to identify all relevant randomized and non-randomized controlled trials. Studies had to report overall PA as the main outcome to be eligible for inclusion in the review. The main outcomes of the studies is a quantified measure of overall PA. Additionally, all studies had to report sex/gender disaggregated overall PA at baseline and/or follow up and/or explain how they dealt with sex/gender during outcome analysis (i.e., sex/gender adjusted analyses) and/or report that there were no differences in the outcome when looking at sex/gender. PRISMA guidelines were followed. Two authors independently screened studies for eligibility and assessed the risk of bias. Semi-quantitative analyses were conducted to evaluate intervention effects, taking into account the extent to which studies have considered sex/gender aspects. To evaluate sex/gender considerations in primary studies, a newly developed sex/gender checklist was used. The study was registered previously (registration number CRD42018109528).

**Results:**

In total, 97 articles reporting 94 unique studies with 164 outcomes for overall PA were included in the present review. Average sample size was 829 participants, ranging from five to 9839. Participants’ ages ranged from three to 19 years. Our review shows that overall 35% of PA outcomes had significant effects in increasing overall PA of children and adolescents. Not including single sex/gender studies, 105 out of 120 PA outcomes resulted in same intervention effects for boys and girls. The interventions reported to have similar effects on PA outcomes for boys and girls showed higher quality of reporting sex/gender aspects of *measurement instruments, participant flow* and *intervention content and materials* than PA outcomes with effects only in boys or only in girls. Overall, consideration of sex/gender aspects in intervention studies is low.

**Conclusions:**

There is still a need to address sufficient consideration of sex/gender aspects in developing and implementing interventions in the context of PA.

## Background

Physical activity (PA) provides numerous health benefits for boys and girls across all ages [1]. Regular PA has preventive effects on the prevalence of overweight, cardiovascular disease, diabetes, hypertension, cancer, depression, and anxiety disorders [2, 3]. Furthermore, as patterns of PA established in childhood tend to continue into adulthood [4], an active lifestyle earlier in life may protect against inactivity and chronic diseases in later life.

The World Health Organization (WHO) recommends that children and adolescents participate in at least 60 min of moderate-to-vigorous PA daily [5]. It is possible to fulfil this recommendation via several shorter bouts of PA throughout the day. Therefore, to evaluate whether children and adolescents achieve these recommendations, it is important to consider cumulative daily (overall) PA levels.

Despite the positive effects of an active lifestyle, suboptimal levels of PA have been observed in children, adolescents, and adults worldwide [6]. Only 27 to 33% of children and adolescents meet the WHO recommendations [7]. PA participation and engagement varies over the life course and there is a notable decline in PA during the transition from childhood to adolescents [8]. Recent analysis of healthy girls and boys ages two to 18 years showed an average decline of 6 min of moderate-to-vigorous PA per day every year [9]. Lower PA levels are reported consistently for girls compared to boys [10] with 85% vs. 78% not meeting WHO PA recommendations [11]. The PA differences between girls and boy are greatest for vigorous PA, less marked for moderate PA, and do not exist for light PA [12]. In addition, types of activity are highly gendered. For example, a recent Norwegian study reported that girls tended to participate in dancing, gymnastics, exercising to music, jumping, or rope skipping whereas boys participated more frequently in team handball, climbing, swimming/water play, mountain hiking, or soccer [13].

Sociocultural norms play an important role in shaping PA preferences among boys and girls, especially with regard to overall PA [14, 15]. The expectancy-value theoretical framework [16, 17] assumes two core characteristics influence behaviour: individuals’ beliefs about expectancies for success and subjective task value, each linked to a broad array of psychological and social/cultural determinants [18]. Recent research suggests that differences in self-efficacy or perceived physical competency and the value attached to PA contribute to gender differences in overall PA participation [19]. In particular, gender identity and sex/gender-based sport stereotyping may affect the amount of perceived competence and subjective value [20, 21]. These non-biologic determinants can be attributed to gender. As sex and gender are intertwined we use the term sex/gender throughout this article.

To date, other than identifying differences in overall PA levels of girls and boys, sex/gender has not been widely considered in systematic reviews. No appropriate guidelines encompassing the implementation and assessment of the effectiveness of sex/gender inclusivity in reviews in the context of overall PA promotion exist. A cross-sectional methods study on reporting sex/gender considerations in systematic reviews of diverse topics demonstrated that less than 30% of reviews reported on sex/gender in the results section [22]. A scoping review of interventions that promote objectively measured overall PA in children indicated that all interventions reported the numbers of participants who were boys or girls at baseline [23]. Nevertheless, the authors did not consider and report how sex/gender was considered in the delivery of the intervention. It would, therefore, be useful to evaluate sex/gender aspects of intervention studies more comprehensively in systematic reviews.

The main objective is to draw on findings of a larger systematic review of sex/gender, PA and sedentary behaviour among children and adolescents to evaluate the effects of interventions on children’s and adolescents’ overall PA and to appraise the extent to which the studies have taken sex/gender into account. Furthermore, the aim is to examine whether the impact of interventions is gendered. To reach this aim, all primary studies included in the review have been assessed using a newly developed sex/gender checklist that builds on prior tools [24].

## Methods

The current study is part of the collaborative genEffects project that evaluates the effects of interventions on girls’ and boys’ PA and sedentary behaviour. The genEffects systematic review on sex/gender is reported according to the PRISMA guidelines ([25], Additional file 1). This part of the genEffects systematic review focuses on interventions to promote overall PA in children and adolescents and, therefore, only primary studies reporting on overall PA as the main outcome were included. Overall PA was defined by a measurement of activity during waking hours of children and adolescents and may have included PA of a specific intensity (e.g., light, moderate or vigorous PA) or all intensities [26]. The protocol for the genEffects project has been published previously [24] and is also registered (ref CRD42018109528). There were no protocol amendments except the GRADE framework was not used due to qualitative analyses of data.

### Search strategy and eligibility criteria

For the genEffects systematic review, a comprehensive literature search was conducted using eleven electronic databases (Cochrane Central Register of Trials (CENTRAL); U.S. National Library of Medicine (clinicalTrials.gov); Ovid Embase; Epistemonikos; EBSCO Eric; WHO International Clinical Trails Registry Platform (ICTRP); Ovid MEDLINE; ProQuest Dissertations & These Global; EBSCO PsycINFO; EBSCO SPORTDiscus; Clarivate Web of Science (Science Citation Index Expanded and Conference Proceedings Index-Science; CPCI-S)) in August 2018. The search strategy was based on Cochrane standards and is included for Ovid MEDLINE as Additional file 2.

Included intervention studies met the following criteria:

1. Participants: healthy children and adolescents with an average age within the range of 3 to 19 years
2. Intervention: aim of intervention has to be promotion of overall PA
3. Study design: randomized controlled trials (parallel group or cluster-randomized) and controlled trials
4. Comparator: active control group, other than PA or sedentary behaviour, or control group with no intervention
5. Outcome: overall PA assessed by any type of measure (subjective/objective); additionally, all intervention studies had to (1) report sex/gender disaggregated PA, at baseline and/or follow up, and/or (2) explain how they dealt with sex/gender during outcome analysis (i.e., sex/gender adjusted analysis), and/or (3) report that there were no differences in the outcome when looking at sex/gender
6. Publication: English language peer-reviewed journal articles published after year 2000

In order to base the results of the systematic review on current activities, only studies published after the year 2000 were included.

### Study selection and data extraction

Study selection for the genEffects systematic review was performed by two independent reviewers using Covidence systematic review software (Veritas Health Innovation, Melbourne, Australia. Available at www.covidence.org). After de-duplication, titles and abstracts were screened, and articles of potential or indeterminate relevance retrieved for full text screening against eligibility criteria. All conflicts were resolved by a third reviewer.

For each included study, study details were extracted using a piloted data extraction form. Data extraction covered information about general study characteristics (country, design, name of intervention program), sample size for intervention and control groups stratified by sex/gender and dropout rates, details about intervention content of the intervention and control groups as well as intervention approaches and settings. Additionally, extraction forms contained information about interventions’ main outcomes, measurement points and instruments, and statistical approaches including confounders taken into account. This information was necessary to analyse the effectiveness of the interventions aiming to promote overall PA. For additional information, study protocols and supplementary materials were used and in case of missing information, the author(s) of the articles were contacted (maximum two contact attempts).

### Quality assessment and risk of bias

Risk of bias was carried out independently by two reviewers using the Cochrane risk of bias tool for randomized trials, version 1 [27]. Using the seven domains of the tool, primary studies were assessed for selection, performance, attrition, detection, reporting, and ‘other’ bias. For ‘other’ bias, we assessed baseline differences between intervention and control arm as well as seasonal differences in measurement points. Each domain was judged as ‘low’, ‘high’ or ‘unclear’ risk of bias, with the last category indicating either lack of information or uncertainty about the potential bias. Discrepancies were resolved through discussion or adjudication by a third reviewer. Review Manager 5 (RevMan 5) [28] software was used to assess the overall risk of bias.

### Sex/gender assessment

To assess the degree to which sex/gender was considered in the included studies, a newly developed sex/gender checklist was used [24]. The sex/gender checklist had been specially developed to rate the degree to which sex/gender aspects have been considered in intervention studies to promote PA or to reduce sedentary behavior. The checklist consists of ten items analysing sex/gender considerations in five categories: background and concepts, study design, intervention planning and delivery, presentation of findings, and interpretation of findings. The items were rated broadly as: ‘not relevant’, ‘basic’, ‘detailed’ or ‘no information provided’. The rating ‘not relevant’ was applied to studies that recruited only boys or only girls for items that were considered less applicable to single sex/gender studies (e.g., provision of sex/gender-disaggregated data for participant flow). The additional grade ‘poor’ was used for the item *definition and use of sex/gender terminology* if sex and gender terminology were used interchangeably within the included articles.

### Data synthesis and statistical analyses

We were unable to conduct meta-analysis as planned [24] due to the heterogeneity (heterogeneous methodologies and outcome measurements) of studies. As only a small subset of all included studies were homogeneous enough to consider meta-analysis, we chose not to combine data. A semi-quantitative analysis was conducted to analyse if the sex/gender-related effects of the included intervention studies were related to the ratings of the sex/gender checklist. Some studies reported more than one outcome for overall PA (e.g., light PA and moderate PA) with different effects with regard to sex/gender for different PA outcomes. Thus, we conducted the analysis on the level of the PA outcomes. Due to missing statistical data in about one third of all included primary studies (e.g., reporting only ‘not significant’ as a result), we were not able to analyse PA outcomes that show an effect in the same direction together. Instead, the study results were divided into three groups: (1) PA outcomes with same/similar significant intervention effects for boys and girls; (2) PA outcomes with same no significant intervention effects for boys and girls; and (3) PA outcomes with different intervention effects for boys and girls. Studies that reported more than one PA outcome with different sex/gender-related effects were assigned to more than one of these three groups (see Additional file 5). In every group for all PA outcomes, sex/gender considerations were specified by calculating sum of ratings for ‘detailed’, ‘basic’, ‘no information provided’, ‘poor’, and ‘not relevant’ for every item of the checklist and by calculating the average number of each rating per grade over all studies in each of the three groups. By applying these analyses, we were able to compare the degree of sex/gender consideration between studies that were or were not effective for both, boys and girls, with studies that revealed different effects for boys and girls, respectively. For single sex/gender studies we compared PA outcomes that were effective with others that were not.

## Results

### Study selection (flow chart)

**Fig. 1:**
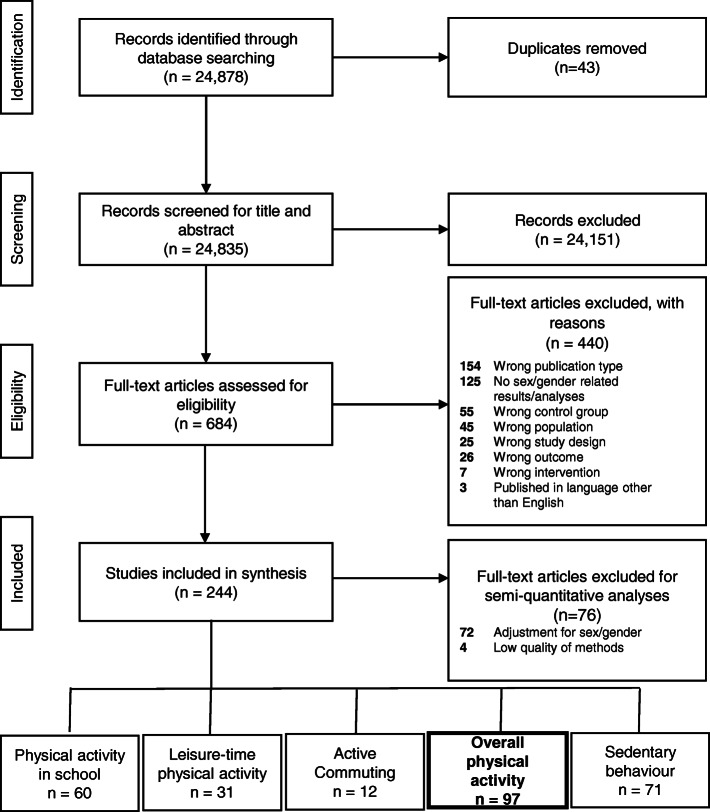
PRISMA Flow Diagram

In total, 97 articles reporting 94 unique studies with 164 outcomes for overall PA were included in this analysis. Originally, in the genEffects systematic review we identified 24,878 references through the electronic database search leading to the inclusion of 244 articles reporting 217 unique studies (Fig. 1).

### Characteristics of included studies and study participants

A table including all relevant characteristics of included studies is presented in Additional file 3. Duration of included studies ranged from 1 week [29] to 6 years [30] excluding follow-up durations. In 58 studies PA was objectively assessed (e.g., accelerometer, pedometer), in 31 subjectively (e.g., diary, questionnaire) and eight studies used both, objective and subjective measurements. Average sample size was 829 participants, ranging from five [31] to 9859 [32]. Control groups of 87 studies received no intervention and continued usual practice. Control groups of ten included studies received information about electricity and energy, support for improving types of foods, child’s safety information or intervention to practice abstinence and to use condoms. Therefore, all interventions of control groups did not aim to improve PA in every respect. With regard to the five settings (school, home, community, child care/kindergarten, health care), 75 studies were conducted in schools, nine studies at home, five studies in communities, three studies in child care/kindergartens and two in the health care setting. Additionally, three studies were not setting oriented. Most included studies (84 studies) used behavioral and social approaches, 55 studies were delivered with campaign and informational approaches and 18 studies used policy and environmental approaches. Overall, 50 were comprised with multicomponent (i.e., more than one approach) approaches. Of the 94 included studies, 50 used cluster randomized designs, 24 were parallel group randomized trials, 19 were (non-randomized) controlled trials and one used cluster control design. Overall, 34 studies reported sex/gender disaggregated data, 13 studies analysed sex/gender within an interaction, 25 studies reported no significant sex/gender differences without reporting the intervention effect (‘tested’) and 25 studies were single sex/gender studies (20 studies only including girls and five only boys). No studies enrolled or identified gender-diverse participants.

### Risk of bias

**Fig. 2:**
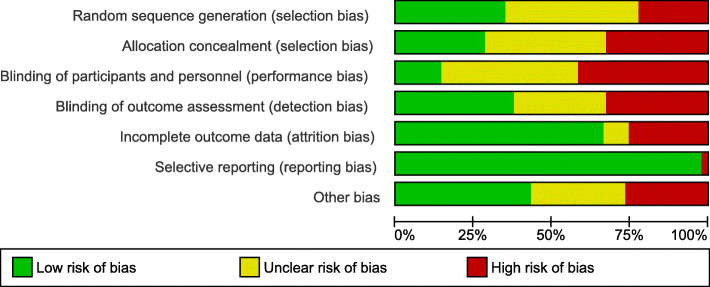
Risk of bias of included studies

Overall 77% of included studies were judged to be at high risk of bias for at least one domain (Fig. 2, Additional file 4). The domain rated as having the lowest risk of bias was selective reporting with 98% of studies at low risk. Random sequence generation was assessed to be at low risk of bias in 35% of studies, allocation concealment was judged at low risk for 29% of studies. 36% of studies reported adequate blinding of outcome assessment and 66% of studies were judged low risk for incomplete outcome data. The risk of bias domain that was judged to have the largest number of high risk studies was blinding of participants (43%). The majority of high risk judgements (27%) of the ‘other’ domain were caused by baseline imbalance of outcome variables or seasonal differences in measurement points.

### Sex/gender checklist

**Fig. 3:**
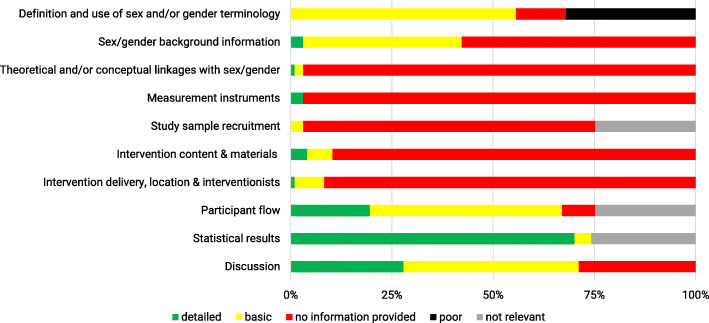
Results of sex/gender checklist

The results of the sex/gender assessment are presented in Fig. 3. We found that 32% of studies were judged to be ‘poor’ for at least one item of the sex/gender checklist. In most studies (96%), at least one sex/gender item was rated as ‘basic’ and in 86% of studies, at least one item was rated as ‘detailed’. The study with the strongest consideration of sex/gender was judged ‘basic’ or ‘detailed’ in eight out of ten items [33] and the study with the lowest consideration of sex/gender did not receive a single rating of ‘basic’ or ‘detailed’ for any of the items [34]. ‘Detailed’ reporting of sex/gender aspects was mostly realized in the statistical results section (70%). The majority of ratings were ‘no information provided’ for *sex/gender background information regarding the research question* (58%), *theoretical and/or conceptual linkages with sex/gender* (97%), *measurement instruments* (97%), *study sample recruitment* (72%), *intervention content and materials* (90%), and *intervention delivery, location and interventionist* (92%). *Definition and use of sex and/or gender terminology* (56%), *participant flow* (47%), and *discussion* (43%) were mostly reported ‘basic’. Overall, across all items ‘no information provided’ (55%) was the most frequent rating. Nevertheless, 21 and 13% of all ratings were ‘basic’ or ‘detailed’, respectively.

### Intervention effectiveness in terms of sex/gender

**Fig. 4:**
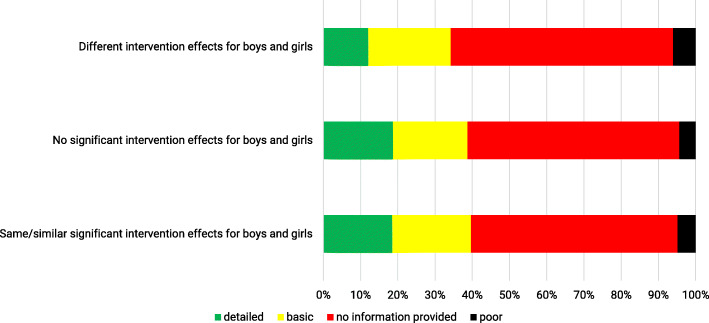
PA outcomes with same/similar effects in girls and boys (with or without significant intervention effects) compared to PA outcomes with different intervention effects for boys and girls

We analysed the relation of intervention effects for each outcome for overall PA with regard to sex/gender by considering the results of the sex/gender checklist which indicated the extent with which studies have taken sex/gender into account (see Additional file 5). For 35 PA outcomes significant intervention effects were found with no differences between boys and girls and in 70 PA outcomes in both boys and girls, no significant intervention effects were reported. Thus, for these 105 PA outcomes no differences in girls and boys were observed. Additionally, 15 PA outcomes revealed different intervention effects in boys and girls. Qualitative analyses considering the sex/gender checklist showed, that there were no differences in how often considerations of sex/gender were rated as ‘poor’, ‘basic’ or ‘no information provided’ in PA outcomes with regard to their results regarding differences or similarities in intervention effects between boys and girls (Fig. 4). Nevertheless, in PA outcomes with similar intervention effects for boys and girls (with or without significant increases of overall PA; *N* = 105), there was a higher amount of ‘detailed’ reporting of the sex/gender checklist (M_detailed_ = 1.8 and 1.5, respectively) compared to PA outcomes with different intervention effects for boys and girls (M_detailed_ = 1.3). In particular, PA outcomes with no differences in intervention effects between boys and girls were more often rated as ‘detailed’ with regard to *measurement instruments, participant flow* and *intervention content and materials.*

### Sex/gender related analyses of single sex/gender studies

Overall, 44 PA outcomes were included in our semi-quantitative analyses of single sex/gender studies. In detail, 23% of single sex/gender PA outcomes revealed a significant intervention effect. When considering all 44 PA outcomes, PA outcomes without significant intervention effects received a ‘detailed’ rating (M_detailed_ = 0.7) more often than PA outcomes with significant intervention effects (M_detailed_ = 0.4). The four items *sex/gender background information, theoretical and/or conceptual linkages with sex/gender, intervention content and materials* and *discussion* were more often reported as ‘detailed’ in PA outcomes without significant intervention effects (see Additional file 5: Summary of all tables).

## Discussion

The aim of this portion of a larger systematic review was to evaluate the effects of interventions on children’s and adolescents’ overall PA in both boys and girls, and to appraise the extent to which the studies have taken sex/gender into account. This review included 94 studies with 164 PA outcomes included measuring a wide range of PA outcomes by any type of measure (subjective/objective). Our review shows that in most PA outcomes the same or similar intervention effects were observed for boys and girls (105 out 120 PA outcomes). The quality of reporting sex/gender aspects captured by applying the newly developed sex/gender checklist was low.

Overall PA is too low in many children and adolescents [6] and thus, improving overall PA is an important concern for both boys and girls. However, as overall PA levels are especially low in girls [35], interventions should contribute to the reduction, or at least not increase, the gradient of sex/gender inequalities in overall PA in childhood and adolescence. In summary, the review revealed that only one third of the outcomes showed improvements in overall PA leading to the conclusion that most interventions failed to increase overall PA. However, this review also identified that most successful interventions were effective for both boys and girls. Furthermore, studies reporting the same or similar effects of PA interventions in boys and girls were more often rated as ‘detailed’ with regard to sex/gender consideration across all items of the checklist when compared with studies reporting different effects. This might suggest that considering sex/gender during intervention planning, development, delivery and analyses increased the likelihood of impact for all. In particular, the items measurement instruments, participant flow, and intervention content and materials were taken into account more strongly in interventions with outcomes with equal effectiveness.

The application of measurement instruments that are sex/gender invariant is important. For example, it has been reported that the Yamax pedometer underestimated the number of steps at slower walking speed. Consequently, lower step counts of girls could be a result of underestimation because girls tend to have smaller stride length, resulting in slower walking speeds [36]. To minimize bias arising from measurement used, it is necessary to consider sex/gender specific characteristics (e.g., weight, height or BMI). Sigmund et al. [33] used relative energy expenditure values for group comparison of boys and girls with different body weights, a measure without apparent gender bias, and found similar intervention effects for boys and girls.

Conclusions about effectiveness should make allowance for participant flow. For conducting sex/gender-based analyses it is important to take into account and to report on the flow of participants according to sex/gender (e.g., recruited, enrolled, completed). This has not been done in 80 % of the included studies as the ratings of our sex/gender checklist revealed. For example, dropout rates (as one indicator of participant flow) from sports participation have been shown to be higher in girls compared to boys. Therefore, sex/gender distribution might be equal for recruitment but not for post or follow-up measurement [37]. As a best practice example out of the included studies in this review, Beets et al. [38] presented the number of participants for baseline, post-intervention and follow-up disaggregated with regard to sex/gender.

Additionally, intervention content and materials should be gender-sensitive to address all participants. As an example, Pardo et al. [39] stated that their intervention program attempted to address the specific interests and needs of boys and girls (e.g., by encouraging them to express their opinions and offer suggestions during the tutorial session), while offering an overall program strategy that was similar for boys and girls. Such considerations may enhance the applicability of interventions for all groups regardless of sex/gender.

For single sex/gender studies, only 23% of PA outcomes showed significant intervention effects (14.3% of PA outcomes for boys and 24.3% of PA outcomes for girls). Interventions with PA outcomes without a significant intervention effect were more often rated as ‘detailed’ with regard to sex/gender consideration. This finding is surprising as interventions in which sex/gender was considered more strongly might be anticipated to be more effective. It is possible that ineffective interventions were reported more precisely with regard to sex/gender consideration than effective ones to overcome potential criticism on the concept and conduct of ineffective interventions [40, 41]. Additionally, the ineffectiveness of most single sex/gender studies could be explained by considering previous research indicating that girls and boys tended to accrue more moderate-to-vigorous PA (MVPA) in coeducational than in unisex classes [42, 43]. Average percentages of physical education time spent in MVPA in coeducational classes is higher than those recorded in unisex classes [43]. Girls and boys reported that they have more fun and a higher social motivation in coeducational classes compared to unisex classes [44]. Nevertheless, as shown in another review on equity effects of children’s physical activity interventions [23], there is no clear evidence on comparative effectiveness of targeted interventions focussing on a specific high-risk subgroup (like girls) and universally targeted interventions. Thus, further research is needed to understand if targeted (intervention with tailored intervention content) or non-targeted interventions are more effective [23] and whether single sex/gender interventions can be effective.

### Implications for research and practice

There is a need to address the inconsistent use of terms sex and gender, the insufficient consideration of sex/gender in developing and implementing interventions, and the lack of robust sex/gender analysis in PA intervention studies. This review demonstrates a need for continued efforts to improve appropriate consideration and reporting of sex/gender during all steps of intervention planning, development, delivery and analysis. Although a variety of initiatives (e.g., Canadian Institutes of Health Research, the Gender Policy Committee of European Association of Science Editors) have attempted to increase the degree to which sex/gender is considered in studies, no appropriate guidelines encompassing sex/gender in interventions and systematic reviews in the context of overall PA exist [45–47]. It is important to consider sex/gender aspects to reduce any sex/gender gap in terms of overall PA. The newly developed sex/gender checklist can help researchers by applying the sex/gender items of the checklist during the development, implementation, and appraisal of overall PA promotion programs. For further research, we recommend identifying and analysing potential moderators such as age, different intervention contents, settings, or methods, different types of overall PA outcomes, cultural or regional location etc. that could have an impact on the effects of the interventions. Finally, to assess the strength of a body of evidence and to carry out the relationship between the results of interventions and the risk of bias, it is advisable for further studies to consider risk of bias in the data synthesis approach (e.g., conduct sensitive analysis and exclude high risk of bias studies from the analysis).

### Strengths and limitations

To our best knowledge our systematic review and semi-quantitative analysis is the first to systematically assess how sex/gender aspects are considered in interventions promoting overall PA in children and/or adolescents. No previous review appraised the extent to which the studies have taken sex/gender into account with a comprehensive checklist and systematically analysed the effectiveness with regard to sex/gender. Furthermore, through our inclusive approach to PA promotion activities, which was not limited to only behavioural and cognitive strategies, we were able to highlight a range of different programmes to improve overall PA in children and/or adolescents. Another strength of the systematic review was using the PRISMA statement to improve the reporting quality.

However, this work has also some limitations. The review is limited to English language articles and did not include studies published in other languages. Furthermore, the research was limited to peer-reviewed journal articles and thus, results of other intervention studies published in other types of literatures were excluded. Regarding the considerations of sex/gender aspects in the primary studies, we were not able to differentiate if these aspects were neglected or just fragmentary or insufficiently reported. However, this can lead to bias and undervaluation of sex/gender considerations in primary studies. It is also worth mentioning, that conclusions should be interpreted carefully because of inability to conduct a meta-analysis because of the heterogeneity of studies. We conducted semi-quantitative analyses using the ratings of the sex/gender checklist without taking their relative weight into account, because until now no theoretical assumption about the weight of the items exists. Additionally, based on the available primary data we were not able to determine if the interventions contributed to gender equity. We just analysed if boys and girls benefited similarly from the intervention regardless of their starting levels of overall PA. Thus, even if they benefited equally at the end of the intervention there can still be unequal levels of overall PA. Finally, our work here is also limited to focusing on the binary characterisation of gender (boys and girls) because none of the included studies included gender diverse participants.

## Conclusion

Despite low overall PA levels in children and adolescents, and different levels of overall PA in boys and girls, the current systematic review confirms that sex/gender aspects have rarely been considered in interventions aiming to increase children’s and adolescents’ overall PA. Nevertheless, most interventions were similarly effective in boys and girls. The findings of this review are of interest to health promoters as well as researchers and policy makers who put effort in promoting overall PA while simultaneously fostering sex/gender equity.

## Supplementary information

**Additional file 1.** PRISMA Checklist; Description of data: Reporting checklist for systematic reviews and meta-analyses (PRISMA).

**Additional file 2.** MEDLINE search strategy; Description of data: search strategy based on Cochrane standards for Ovid MEDLINE.

**Additional file 3.** Overview of included studies; Description of data: table including all relevant characteristics of included studies.

**Additional file 4.** Risk of bias summary table; Description of data: risk of bias summary table.

**Additional file 5.** Intervention effects in relation to considerations of sex/gender in the included studies; Description of data: relation of intervention effects for each outcome for overall PA with regard to sex/gender by considering the results of the sex/gender checklist which indicated the extent with which studies have taken sex/gender into account.

## Data Availability

Data of the genEffects project as well as the sex/gender checklist are available on request from the project leader, Prof. Dr. Yolanda Demetriou (yolanda.demetriou@tum.de).

## Abbreviations

PA: Physical activity
WHO: World Health Organization
