# Supplementary material for: A sex/gender perspective on interventions to promote children’s and adolescents’ overall physical activity: results from genEffects systematic review
Source: BMC Pediatr. 2020 Oct 10;20:473. doi: 10.1186/s12887-020-02370-9 (PMC7547493; doi:10.1186/s12887-020-02370-9)
Supplement: Supplementary file 2 — Additional file 2. MEDLINE search strategy; Description of data: search strategy based on Cochrane standards for Ovid MEDLINE. [file 12887_2020_2370_MOESM2_ESM.docx]

**Additional file 2: MEDLINE search strategy**

From: Interventions on children’s and adolescents’ physical activity and sedentary behaviour: protocol for a systematic review from a sex/gender perspective

|  |  |
| --- | --- |
| 1 | Adolescent/ (1879830) |
| 2 | exp Child/ (1783242) |
| 3 | (adolesc$ or boy? or child$ or girl? or juvenile? or kid? or school$ or school age$ or student? or teen$).ti,kf. (1090760) |
| 4 | (adolesc$ or boy? or child$ or girl? or juvenile? or kid? or school$ or school age$ or student? or teen$ or youth?).ab. /freq = 2 (908853) |
| 5 | or/1-4 (3245722) |
| 6 | exp Motor Activity/ (259691) |
| 7 | exp Exercise/ (168655) |
| 8 | exp Exercise Therapy/ (43456) |
| 9 | exp Recreation/ (187698) |
| 10 | exp *Sports/ (113371) |
| 11 | Physical Exertion/ (55439) |
| 12 | exp Physical Fitness/ (26257) |
| 13 | exp “Play and Playthings”/ (12587) |
| 14 | (active or activities or activity or aerobic? or athletic? or badminton or baseball or basketball or bicycl$ or bike? or biking or boxing or cardio or cricket or cycling or dance or dancing or exercis$ or fitness or football or gymnastic? or handball or hockey or jogging or jiu jitsu or judo or jujitsu or karate or playground? or rugby or running or soccer or sport? or swim$ or tennis or training or volleyball or walk? or walking or yoga).tw,kf. (4104009) |
| 15 | physical$ activ$.tw,kf. (97509) |
| 16 | Sedentary Lifestyle/ (6980) |
| 17 | Television/ (12915) |
| 18 | (gaming or television or tv or video game? or videogame?).tw,kf. (27953) |
| 19 | or/6-18 (4250380) |
| 20 | intervention?.ti. and 19 (26682) |
| 21 | ((amount? or effect? or effectiveness or encourag$ or evaluat$ or impact? or improve$ or improving or increase? or increasing or intervention? or modif$ or promot$) adj3 (activity level? or exercise or fitness or mobility or physical activit$ or step$)).ti,kf. (24388) |
| 22 | ((amount? or effect? or effectiveness or encourag$ or evaluat$ or impact? or improve$ or improving or increase? or increasing or modif$ or promot$) adj4 (activity level? or exercise or fitness or mobility or physical activit$ or step$)).ab. (131764) |
| 23 | ((avoid$ or curb$ or decreas$ or discourag$ or effect? or effectiveness or eliminat$ or evaluat$ or impact? or modif$ or prevent$ or reduc$) adj3 (computer$ or inactiv$ or screen-based or screen time or sedentary or sitting or television or tv or video game?)).ti,kf. (4977) |
| 24 | ((avoid$ or curb$ or decreas$ or discourag$ or effect? or effectiveness or eliminat$ or evaluat$ or impact? or modif$ or prevent$ or reduc$) adj4 (computer$ or inactiv$ or screen-based or screen time or sedentary or sitting or television or tv or video game?)).ab. (31431) |
| 25 | or/20-24 (196821) |
| 26 | 5 and 25 (30682) |
| 27 | randomized controlled trial.pt. (466685) |
| 28 | controlled clinical trial.pt. (92572) |
| 29 | randomi?ed.ab. (501162) |
| 30 | placebo.ab. (190972) |
| 31 | clinical trials as topic/ (184490) |
| 32 | randomly.ab. (295330) |
| 33 | trial.ti. (186030) |
| 34 | (allocation or allocated).ab. (91828) |
| 35 | assigned.ab. (212727) |
| 36 | (controlled adj2 (study or trial)).ab. (130228) |
| 37 | control group?.ab. (399548) |
| 38 | ((singl$ or doubl$) adj blind$).ab. (141426) |
| 39 | or/27-38 (1625780) |
| 40 | animals/ not (humans/ and animals/) (4453468) |
| 41 | 39 not 40 (1431749) |
| 42 | 26 and 41 (8753) |
| 43 | limit 42 to yr = “2000 - 2018” (7727) |
