# Supplementary material for: A sex/gender perspective on interventions to promote children’s and adolescents’ overall physical activity: results from genEffects systematic review
Source: BMC Pediatr. 2020 Oct 10;20:473. doi: 10.1186/s12887-020-02370-9 (PMC7547493; doi:10.1186/s12887-020-02370-9)
Supplement: Supplementary file 3 — Additional file 3. Overview of included studies; Description of data: table including all relevant characteristics of included studies. [file 12887_2020_2370_MOESM3_ESM.docx]

**Additional file 3: Overview of included studies**

| **Intervention name**  **Study**  (first author; year of publication, country) | **Design**  (study design, analysis of sex/gender) | **Participants**  (n total; n control; age ± SD; % male) | **PA measurement**  (measure used; outcomes reported) | **Intervention**  (intervention duration; frequency; theoretical grounding) | **Intervention**  (IG; CG) | **Intervention outcome** | **Intervention outcome with regard to sex/gender** |
| --- | --- | --- | --- | --- | --- | --- | --- |
| **n.a.**  Aburto et al.; 2011; Mexico | cluster RCT (2 IG, 1 CG); tested | n=699; n CG=217; age= 10.2 ±0.69y; ♂=47% | objective (pedometer); total step count (steps per day) | 12 months; intervention group 1: 50min/week; intervention group 2: 50min /week; n.a. (theoretical background) | IG1   - awareness raising meetings with staff focusing on:  1. Benefits and importance PA 2. Strategies for increasing PA of students (e.g. allowing time to break from study and moving during class)   IG2   - Session of exercise conducted daily with entire student body. 20min before class began  1. Teacher promotion of activity, use of an "activity box" (contained flash cards with ideas to promote activity) 2. Enhanced follow-up with schools by project staff including visiting schools to support teachers in promotion of activity during recess, answering questions regarding promotion of activity   CG   - one PE class / week with average duration of 39min; no intervention | **Sig. intervention effects (only for IG1)**  controlled for baseline values  IG1:CG; *p*=.03  IG2:CG; *p*=.26 | **Same/similar intervention effects for both** ♂ **and** ♀  Intervention effects did not differ by sex. |
| **Som la Pera ("We are Cool")** Aceves-Martins et al.; 2017; Spain | cluster RCT; dis. | n=393: n CG=162; age= 14.66± 0.77y; ♂=48% | subjective (survey); total PA (% ≥6h PA/week) | 12 months; permanent intervention; healthy lifestyle theory | IG   - Training of adolescent challenge creators on social marketing principles and healthy lifestyle theory - 90min/week, 24 weeks activity design sessions, conducted by health promotion and communication specialists; e.g. increase of PA - Challenge creators present intervention in 11 participating classrooms: explained study, provided social media information, invited peers to provide suggestions for activities - implemented 10 activities over 12 months   CG   - No intervention | **Sig. intervention effects**  time*group; *p*<.01 | **Different effects in** ♂**/**♀**in favour of** ♂  ♂; time*group; *p*<.01  ♀; time*group; *p*=.07 |
| **Midland activity lifestyle and healthy eating in school children (WAVES) study**  Adab et al.; 2018; UK (a) | cluster RCT; dis. | n=2462; n CG= 1328; age= 6.3± 0.3y; ♂=50% | objective (accelerometer); MVPA (% ≥60min/ day) | 12 months; daily (30min; MVPA promotion), 2 day trips (Villa Vitality program), 3 workshops (healthy cooking), permanent (information sheets); n.a. (theoretical background) | IG   - Helping teachers to provide opportunities for additional MVPA during school day - Participation in “Villa Vitality” programme, delivered through sport institution - Healthy cooking workshops - Information sheets to families signposting PA opportunities   CG   - Received resources for ‘Your World, My World’ resource from Oxfam (Oxford, UK), exploring the lives of four children from around the world - And ‘Climate Cops’ resource from Npower (Npower Ltd, Swindon, UK) – a teaching toolkit on electricity and energy | **No sig. intervention effects**  time*group; *p*=.303 | **Same/similar intervention effects for both** ♂ **and** ♀  ♂; time*group; *p*=.832  ♀; time*group; *p*=.749 |
| **Midland activity lifestyle and healthy eating in school children (waves) study**  Adab et al.; 2018; UK (b) | cluster RCT; tested | n=1397; n CG= 735; age= 6.3±0.3y; ♂=51% | objective (accelerometer); MVPA (mins/24hrs, ≥60min/ day), | 30 months; daily (30min; MVPA promotion), 2 day trips (Villa Vitality program), 3 workshops (healthy cooking), permanent (information sheets); n.a. (theoretical background) | IG   - Helping teachers to provide opportunities for additional MVPA during school day - Participation in “Villa Vitality” programme, delivered through sport institution - Healthy cooking workshops - Information sheets to families signposting PA opportunities   CG   - Received resources for ‘Your World, My World’ resource from Oxfam (Oxford, UK), exploring the lives of four children from around the world (www.oxfam.org.uk/education/resources/your-world-my-world) - And ‘Climate Cops’ resource from Npower (Npower Ltd, Swindon, UK) – a teaching toolkit on electricity and energy. | **No sig. intervention effects**  group*time; n.s. (*p*=n.a.) | **Same/similar intervention effects for both** ♂ **and** ♀  Subgroup analyses of sex were consistent with the main analyses and did not change any conclusions. |
| **"ACTIVITAL"** Andrade et al.; 2014; Ecuador | cluster RCT; tested | n=1440; n CG=740 ; age=15.1± 0.7y; ♂=37% | objective (accelerometer); total PA (counts per day, cpm/day), LPA and MVPA (min/day) | 28 months; one chapter every 2 weeks (books), two parental workshops, once (social event), permanent (walking trail and posters); SCT, control theory, TPB | IG   - Reading books with information about PA - Parental workshops to increase activity behavior - Social events, walking trial and posters with information about healthy behaviours   CG   - Standard PE | **No sig. intervention effects**  adjusted for baseline values  total PA (counts per day); IG:CG; *p*=.18  total PA (cpm/day); IG:CG; *p*=.23  LPA; IG:CG; *p*=.32  MVPA; IG:CG; *p*=.08 | **Same/similar intervention effects for both** ♂ **and** ♀  Intervention effect was not different for ♂ and ♀. |
| **Youth Fit 4 Life (YF4L)**  Annesi et al.; 2015; USA | RCT: interaction (2 IG, 1 CG) | n=138; n CG=46; age=9.7 ±0.8y; ♂=49% | objective (accelerometer); MVPA, MPA (min/45min), VPA, LPA (mean min of 3 measurement points) | 3 months (of measurements); frequency unclear; SCT, self-efficacy theory | IG1   - Standard Intervention: counsellors with 5 hours of training; instructor manual, participant workbook, 30-35min PA via non-competitive games   IG2   - Revised intervention: newly designed training of counsellors, strength training component, behavior and nutrition topics were reinforced, 30-35min sessions for PA; 35-40min sessions of MVPA, separate training manual for 5-8 year old and 9-12 year old children   CG   - Typical care | **Sig. intervention effects (only for MVPA and VPA)**  MVPA; mean min; group effects; *p*<.001  MPA; mean min; group effects; *p*=.085  VPA; mean min; group effects; *p*<.001  LPA; mean min; group effects, *p*=.130 | **Same/similar intervention effects for both** ♂ **and** ♀  MVPA; sex*group; *p*=.582  MPA; sex*group; *p*=.354  VPA; sex*group; *p*=.692  LPA; sex*group; *p*=.748 |
| **Girls on the Move (GOTM)**  Bakhoya et al.; 2016; USA | cluster RCT; single sex/gender (♀) | n=181; n CG=64; age= 11.97±0.70y; only ♀ | objective (accelerometer); MVPA, LPA (min/day) | 8 months; 13 postcards per 8 months; n.a. (theoretical background) | IG   - Booster intervention with 13 motivational, individual tailored postcards, enclosed in bright pink envelopes mailed to each girl’s home during postintervention - Each postcards included: fun activity about PA for girls to complete and a motivational, individually tailored message to encourage PA - Messages were tailored based on each girl’s personal responses to iPad survey   CG   - No intervention | **No sig. intervention effects**  LPA; CG; time effects; *p*<.01  LPA; IG; time effects; *p*<.01  MVPA; CG; time effects; *p*<.01  MVPA; IG; time effects; *p*<.01 | **No sig. intervention effects in** ♀ |
| **STEPs-PA**  Beets et al.; 2015; USA | cluster RCT; dis. | n=1627; n CG=864; age= 7.9± 1.8y; ♂=52% | objective; MVPA (≥30min/day; min/day) | 12 months; one workshop (3 hours); system change theory | IG   - Strategies: following schedule of daily programming, allocated time for PA (60min/day), staff PA-related training (workshop for 3 hours)   CG   - No intervention | **Sig. intervention effects**  (OR for intervention)  30min/day MVPA; ♂; OR=2.26 (95%CI=1.35 to 3.80)  30min/day MVPA; ♀; OR=2.85 (95%CI=1.43 to 5.68)  MVPA (min/day), ♂; OR=4.0 (95%CI=2.2 to 5.8)  MVPA (min/day); ♀; OR=2.7 (95%CI=1.3 to 4.2) | **Same/similar intervention effects for both** ♂ **and** ♀  Sig. intervention effects for both ♂ and ♀ |
| **SYM-KEM-Study**  Bhave et al.; 2016; India | CT; dis. | n=865; n CG=53; age=13-14 years; ♂=53% | subjective (questionnaire); total PA (h/day) | 5 years; once a week (increase PA session, health education programme); daily (yoga exercise); once (workshop); n.a. (theoretical background) | IG   - Increasing PA sessions to 6 per week - Training teachers to engage children in daily yoga-based breathing exercise - Offering attractive PA sessions during holidays - Health education programme, with weekly age-appropriate interaction 1-h sessions teach children about importance of healthy life - Workhops for teachers   CG   - No intervention | **No sig. intervention effects**  baseline; IG:CG; *p*<.001  (favoured IG)  posttest; IG:CG; *p*<.001  (favoured IG)  ♂; baseline; IG:CG; *p*<.001 (favoured IG)  ♂; posttest; IG:CG; *p*<.001 (favoured IG)  ♀; baseline; IG:CG; *p*=.05  ♀; posttest; IG:CG; *p*<.001 (favoured IG) | **Same/similar intervention effects for both** ♂ **and** ♀  No sig. intervention effects for both ♂ and ♀ |
| **Challenge!**  Black et al.; 2010; USA | RCT; tested | n=235; n CG= 114; age=13.3± 1.0y; ♂=51% | objective (accelerometer); total PA (cpm), play equivalent PA (min/day) | 12 months; 12 sessions (rap music); SCT | IG   - Rap music video promoting healthy eating and PA - Principles of mentorship (role modelling and support), participatory learning, and goal setting were central to intervention - Setting of PA goals, tracking and evaluating progress, revising goals as necessary - Mentors received training, including motivational interviewing and weekly supervision   CG   - Did not receive a mentor or any contact between baseline and follow-up evaluations | **No sig. intervention effects**  total PA; group*time; *p*=.133  play PA; group*time; *p*=.155 | **Same/similar intervention effects for both** ♂ **and** ♀  Intervention effects were not sig. for ♂ and ♀. |
| **n.a.**  Bryant et al.; 2016; UK | CT; dis. | n=165; n CG=83; age= 8.3±0.4y; ♂=46% | objective (pedometer);  steps (per day) | 6 weeks; one PE session per week; n.a. (theoretical background) | IG   - Replace of 1 PE session (e.g. warm up, instruction time, circuits, cool-down song) by focusing on fundamental movement skills   CG   - Continue with normal PE sessions | **Sig. intervention effects**  ♂; time*group; *p<*.05  ♀; time*group; *p*<.01 | **Same/similar intervention effects for both** ♂ **and** ♀  Intervention effects were significant for ♂ and ♀. |
| **Pathways Study**  Caballero et al.; 2003; USA | RCT; tested | n=1704; n CG= 825; age= 7.6±0.6y; ♂=n.a. | objective (accelerometer); total PA (cpm) | 3 years; 3x30min PE sessions/ week, daily (exercise breaks, guided play during recess); social learning theory | IG   - Classroom curriculum: promoting PA - PE: increase energy expenditure (three 30-min PE sessions/week during school time); exercise break during classroom time; guided play during recess - Family fun nights, workshops; events at school, family packs linked to classroom curriculum   CG   - No intervention | **No sig. intervention effects**  controlled for baseline values  objective PA; IG:CG; *p*=.310 | **Same/similar intervention effects for both** ♂ **and** ♀  No effect differences between ♂ and ♀. |
| **Comprehensive School Physical Activity Program (CSPAP)**  Carson et al.; 2014; USA | CT; dis. | n=351; n CG=120; age=11.2± 0.7; ♂=63% | objective (accelerometer); MVPA (min/day) | 12 months; one 6hr workshop, permanent support; ecological system theory | IG   - 6h experimental workshop where PE teachers received information and skill-based training, develop and action plan to implement a new and achievable PA opportunity - Support for teachers   CG   - No intervention, waiting CG | **No sig. intervention effects**  time*group; n.s. (*p*=n.a.)  ♂; time*group; n.s. (*p*=n.a.)  ♀; time*group; n.s. (*p*=n.a.) | **Same/similar intervention effects for both** ♂ **and** ♀  Intervention effects were not significant for ♂ and ♀. |
| **The Peadiatric Osteoporosis Prevention (POP) study**  Cronholm et al.; 2017; Sweden | CT; dis. | n=194; n CG=88; age=7.8 ±0.6y (♂ IG), 7.9±0.6y (♂ CG), 7.7±0.7y (♀ IG), 7.9±0.7y (♀ CG); ♂=66.4% | subjective (no validated questionnaire); total PA (h/week) | 36 months; daily interventions all school days all school weeks; activity-sate theory | IG   - Extra PE classes: increase PE curriculum of 200min per week; daily 40min classes during 5 school days all school weeks - Ordinary teachers supervised PE classes that included a variety of different activities, such as ball sports, running, climbing and jumping   CG   - No intervention | **Sig. intervention effects**  n.s. differences for ♂ and ♀in IG and CG at baseline before study start (*p*=n.a.)  Baseline after study start 1  ♂; time effects; *p*<.05 (favoured IG)  ♀; time effects; *p*<.05 (favoured IG)  Year 2  ♂; time effects; *p*<.05 (favoured IG)  ♀; time effects; *p*<.05 (favoured IG)  Year 3  ♂; time effects; *p*<.05 (favoured IG)  ♀; time effects, *p*<.05 (favoured IG) | **Same/similar intervention effects for both** ♂ **and** ♀  Intervention effects were significant for ♂ and ♀. |
| **The Chile Study**  Cruz et al.; 2016; USA | RCT; tested | n=665; n CG= 327; age=3-5y; ♂=50% | subjective (questionnaire); engagement in PA behaviours (“often”, “sometimes”, “never”) | 2 years; daily classroom PA; n.a. (theoretical background) | IG   - Physical activity through a classroom curriculum (5-30min), professional development for teachers, family events at centres, take-home materials   CG   - Participated in usual PA practices | **Sig. intervention effect**  group*time; *p*=.04 | **Same/similar intervention effects for both** ♂ **and** ♀  Analyses conducted to assess whether intervention effects were different for ♂ and ♀ did not detect a significant interaction. |
| **n.a.**  Cui et al.; 2012; China | cluster RCT; tested | n=682; n CG= 285; age= 12.6±0.5y; ♂=51.75% | subjective (questionnaire); MVPA (MET 3 min/day) | 7 months; between three times a 90min; SCT, empowerment educational approach | IG   - Four-component peer-led health programme (food choice, PA, SB, carbonated drinks and goal setting) - Learning activities were designed to be conducted in a variety of ways: presentations, video watching, group discussion, games, experiments, lifestyle practice, skit playing, quiz show - Peer leader’s manual to describe the structural activities   CG  No intervention was implemented | **No sig. intervention effects**  baseline; IG:CG; n.s. (*p*=n.a.)  3 months; IG:CG; *p*=.83  7 months; IG:CG; *p*=.94 | **Same/similar intervention effects for both** ♂ **and** ♀  Sex-by-group interaction term showed no significance. |
| **n.a.**  Darabi et al.; 2017; Iran | cluster RCT; single sex/gender (♀) | n=678; n CG=285; age= 12.6±0.5y; only ♀ | subjective (questionnaire); PA behavior (based on theory of planned behavior) | 10 months and 20 days; one educational session (2 parts of 45min), permanent (booklet); TPB | IG   - Four-component peer-led health programme: food choice, PA, SB, carbonated drinks and goal setting - Learning activities via presentations, video watching, group discussion, games, experiments, lifestyle practice, skit playing, quiz show - Peer leader’s manual to describe structured activities   CG   - Did not receive any additional education or experimental material | **Sig. intervention effects**  time*group; *p*<.001 (favoured IG) | **Sig. intervention effects in** ♀ |
| **ToyBox-Intervention**  De Craemer et al.; 2014; Belgium | cluster RCT; dis. | N=472; n CG= 171; age= 4.43± 0.55y; ♂=55.1% | objective (accelerometer); MVPA, VPA, total PA, MPA (% of day) | 6 months; once per week (60min); SEM, behavior change theory | IG   - Interventions implemented by kindergarten teachers; teachers had 2 training sessions (researchers explain goals and material) - ToyBox Teachers’ guide (e.g., background information, definition of PA etc.), classroom activity guides, newsletters, tip-cards, posters, kangaroo hand puppet   CG  no access to ToyBox | **No sig. intervention effects**  MVPA, VPA, total PA, MPA; ♂; time*group; n.s. (*p*=n.a.)  MVPA, VPA, total PA, MPA; ♀; time*group; n.s. (*p*=n.a.) | **Same/similar intervention effects for both** ♂ **and** ♀  Intervention effects were not significant for ♂ and ♀. |
| **The Nutrition and Enjoyable Activity for Teen Girls (NEAT**  **Girls) program**  Dewar et al.; 2014; Australia | cluster RCT; single sex/gender (♀) | n=357; n CG=179;  age= 13.2± 0.5y; only ♀ | objective (accelerometer); VPA, MPA, MVPA (%/day) | 12 months; 40 times a 90min (enhances school sport session); SCT | IG   - School sport sessions, interactive seminars, nutrition workshops, lunch-time PA, PA and nutrition handbooks, parent newsletters, pedometers for self-monitoring, text messaging for social support   CG  no intervention | **No sig. intervention effects**  MPA; time*group; *p=*.24  VPA; time*group; *p*=.87  MVPA; time*group; *p*=.37 | **No sig. intervention effects in** ♀ |
| **''Health Scores!'**  Dubuy et al., 2014 | CT; single sex/gender (♂) | n=545; n CG=268; age=12.57± 1.02y (IG), 12.08± 1.58y (CG); only ♂ | subjective (questionnaire); total PA (min/week) | 4 months; n.a. (frequency, theoretical background) | Intervention   - School-based program: school and classroom activities (e.g. active playgrounds and activity breaks during lessons) - Letters from professional football players reminding the pupils of the importance of regular PA   Control   - Usual care | **No sig. intervention effects**  time*group; n.a. (*p*=n.a.) | **No sig. intervention effects in ♂** |
| **'Healthy Homework' Programme**  Duncan et al., 2011 | cluster RCT; interaction | n=97; n CG= 40;  age=9-11years; ♂=36.82% | objective (pedometer); steps (per day overall and on weekdays) | 1.5 months, 2 times per week; information-motivation-behaviour skills model, theory of reasoned action, TPB, SCT, control theory, operant conditioning | IG   - Each child received homework booklet organized into 5 PA topics - Tasks were designed to encourage parental participation and family involvement - 3 homework options for PA; children were required to complete at least one task per week for PA/week (e.g., walking from and to school) - In-class exercises for three 1.5 hours session each week   CG   - No intervention | **Sig. intervention effects**  steps/day; group*time; *p*=.013 (favoured IG)  steps/weekday; group*time; n.s. (*p*=n.a.) | **Same/similar intervention effects for both** ♂ **and** ♀  steps/day; time*sex; *p*=.310  steps/day; group*sex; *p*=.851 |
| **n.a.**  Dunton et al.; 2007; USA | CT; single sex/gender (♀) | n=146; n CG=67; age=15.10; only ♀ | subjective (3 day PA recall); VPA (% of participants reporting some VPA) | 9 months; five days per week (60min each day); SCT | Intervention   - Class activities included yoga, aerobics, basketball swimming, weight training, hip-hop, dance, soccer, walking, and kickboxing - weekly lectures and discussions addressed topics such as time management, body image, motivation, nutrition, and strength training   Control   - Participants were not provided with any particular instructions with regards to participating in PA | **Sig. intervention effects**  group*time; *p*<.01 (favoured IG) | **Sig. intervention effects in** ♀ |
| **Get Educated! CHANGE!**  Fairclough et al.; 2013; UK | cluster RCT; interaction | n=318; n CG= 152; age=10.6± 0.3y; ♂=n.a. | objective (accelerometer); LPA, MVPA, VPA (min/day) | 5 months; weekly 60min; SCT | IG   - Teachers received 4 hours of training in the delivery of curriculum resource - Worksheets, weekly lesson plans, homework tasks, lesson resources, CD-ROM - Key component: “Move more and sit less”   CG   - No intervention | **No sig. intervention effects**  baseline to post-intervention; adjusted for group and baseline values  MVPA; *p*=.62  VPA; *p*=.86  baseline to follow-up; adjusted for group and baseline values  MVPA; *p*=.52  VPA; *p*=.50 | **Same/similar intervention effects for both** ♂ **and** ♀  Intervention effects were not sig. for ♂ and ♀. |
| **“Fortaleça sua Saúde” program**  Filho et al.; 2016; Brazil | cluster RCT; interaction | n=1085; n CG=637; age=11-18y; ♂=51% | subjective (questionnaire); collective PA/sports, individual PA/sports, strengthening PA, walking, popular games, ride, MVPA (min/week), number of PA’s (per week) | 4 months; 4 hours at the beginning (training), 2 per week a 10-15min (opportunities in school environment), permanent (health education); n.a. (theoretical background) | IG   - Training and activities in general curriculum - PE teacher-specific training - Opportunities in the school environment to engage in PA - Health education in school community (e.g. posters)   CG   - Conventional activities | **Sig. intervention effects (for MVPA and number of PA’s)**  adjusted for baseline values  collective PA; IG:CG; *p*=.081  individual PA; IG:CG; *p*=.822  strengthening PA; IG:CG; *p*=-.862  walking; IG:CG; *p*=.814  popular games, IG:CG; *p*<.001  riding; IG:CG; *p*=.862  other PA; IG:CG; *p*=.500  MVPA; IG:CG; *p*=.032  number of PA’s; IG:CG; *p*<.001 | **Same/similar intervention effects for both** ♂ **and** ♀  collective PA; group*gender; *p*=.381  individual PA; group*gender; *p*=.140  strengthening PA; group*gender; *p*=.132  walking; group*gender; *p*=.814  popular games, group*gender; *p*=.786  riding; group*gender; *p*=.302  other PA; group*gender; *p*=.674  MVPA; group*gender; *p*=.066  number of PA’s; group*gender; *p*=.595 |
| **n.a.**  Ghaffari et al.; 2013: Iran | RCT; single sex/gender (♂) | n=85; n control= 43; age=12.2± 0.8y; only ♂ | subjective (questionnaire); total PA (min/day) | 2 weeks; six training sessions; n.a. (theoretical background) | IG   - Knowledge and attitude sessions (e.g. speech, group discussion, question and answers etc.)   CG   - No intervention | **Sig. intervention effects**  Before intervention; IG:CG; *p*=.155  immediately after intervention; IG:CG; *p*<.001 (favoured IG)  1 month after intervention; IG:CG; *p*<.001 (favoured IG) | **Sig. intervention effects in** ♂ |
| **Thao-Child Health Program (TCHP)**  Gomez et al.; 2018; Spain | RCT; dis. | n=2086; n control= 1112; age=14.3± 0.8y; ♂=52% | subjective (questionnaire); total PA (PAQ-C-Score) | 2 years; permanent; attitude-social influence self-efficacy model | IG   - World Health Day events - Parent conference - Physical activity conference - Community-based activities   CG   - No intervention | **No sig. intervention effects**    ♂; group*time; *p*=.98  ♀; group*time; *p*=.98 | **Same/similar intervention effects for both** ♂ **and** ♀  Intervention effects were not significant for ♂ and ♀. |
| **Interactive Multimedia for Promoting Physical Activity (IMPACT)**  Goran et al.; 2005; USA | RCT; dis. | n=112; n CG=59;  age=14-15y; ♂=41% | objective (accelerometer); total PA (cpm), LPA, MPA, VPA, MVPA (%/day) | 8 weeks; weekly intervention; SCT, SEM | IG   - Interactive CD-ROM; 4 classroom and 4 homework assignments - Eight CD-Rom lessons (45min), four classroom lessons (45min), 4 family-based assignments (45min)   CG   - Two control schools received educational CD-ROMS not related to health outcomes | **Sig. intervention effects**  models adjusted for baseline values  total PA; group effects; *p*=.004  LPA; group effects; *p*=.052  MPA; group effects; *p*=.03  VPA; group effects; *p*<.001  MVPA; group effects; *p*=.02 | **Different effects in** ♂**/**♀**in favour of** ♂  total PA; sex*group; *p*=.04 (favoured ♂)  LPA; sex*group; *p*=.05  MPA; sex*group; *p=.*03 (favoured ♂)  VPA; sex*group; *p*<.001 (favoured ♂)  MVPA; sex*group; *p*=.02 (favoured ♂) |
| **GreatFun2Run**  Gorely et al.; 2011; UK | CT; dis. | n=421; n control=215;  age=8-10y; ♂=49% | objective (pedometer, accelerometer) steps (per day), MVPA (min/day) | 10 months; 2 hours classroom activities per week; SCT | IG   - Classroom activities: reinforce children’s learning and encouraged them to reflect on their PA levels - CD-ROM learning and teaching resource for teachers; interactive website for pupils, teachers and parents - PA events, outreach to parents and locals   CG   - Usual PE and health curriculum | **No sig. intervention effects**  MVPA and steps; group*time; n.s. (*p*=n.a.)  MVPA and steps; ♂; group*time; n.s. (*p*=n.a.)  MVPA and steps; ♀; group*time; n.s. (*p*=n.a.) | **Same/similar intervention effects for both** ♂ **and** ♀  Intervention effects were not sig. for ♂ and ♀. |
| **The Physical Activity as Civil Skill Program**  Grasten et al.; 2017; Finland | CT; interaction | n=143; n CG=97;  age=14.48± 0.99y; ♂=n.a. | subjective (questionnaire); MVPA (days per week with ≥60min MVPA) | 12 months; permanent intervention; Achievement goal theory | IG   - Extended break (30min in additional to usual breaks) - Access to fitness hall - Supervised ball games (i.e. soccer) - Equipment supply   CG   - Usual program delivery | **No sig. intervention effects**  time*group; *p*=.551 | **Same/similar intervention effects for both** ♂ **and** ♀  baseline; IG; gender differences; *p*=.04  post-test; IG; gender differences; *p*=.12  baseline; CG; gender differences; *p*=.042  post-test; CG; gender differences; *p*=.17 |
| **TEAM Mississippi Project (including intervention elements from PATHWAYS and CATCH)**  Greening et al. 2011; USA | RCT; interaction | n=450; n CG=246;  age= 8.34± 1.3y; ♂=48% | subjective (questionnaire); score of PA (ranged from 0-21) | 8 months; monthly PA events; social learning theory | IG   - Involving teachers, the state’s education department, health professionals from academic institutions, primary caretakers - Elements from established school-based programs (Pathways and CATCH project) - PA events designed to promote PA   CG   - Followed state’s standard health curriculum (included didactic nutrition education. health information incorporated into academic lessons; weekly PE classes) | **Sig. intervention effects**  time*group; *p*=.04 | **Same/similar intervention effects for both** ♂ **and** ♀  time*group*gender; *p*>.10 |
| **HEalth in Adolescents (HEIA) study**  Grydeland et al., 2013 | RCT; dis. | n=700; n CG= 485;  age=11.2± 0.3y (CG), 1.2±0.3y (IG); ♂=43.0% | objective (accelerometer); total PA (mean cpm), LPA, MVPA (min/day) | 20 months; once per week or permanent; SEM | IG:   - Team of teachers involved to promote healthy development - Yearly kick-off meeting with teachers to ensure they knew rationale and were familiar with intervention components - Teachers received external support in form of short monthly e-mail from HEIA-study-group - Parental fact sheets with information about screen time   CG:   - No intervention | **Sig. intervention effects (for total PA)**  total PA; time*group; *p*=.05  LPA; time*group; *p*=.33  MVPA; time*group; *p*=.45  total PA; ♂; time*group; *p*=.35  total PA; ♀; time*group; *p*=.03 (favoured IG)  LPA; ♂; time*group; *p=*.63  LPA; ♀;time*group; *p*=.33  MVPA; ♂; time*group; *p*=.77  MVPA; ♀; time*group; *p*=.13 | **Different effects in** ♂**/**♀**in favour of** ♀  total PA; ♂; time*group; *p*=.35  total PA; ♀; time*group; *p*=.03 (favoured IG) |
| **ZAMZEE**  Guthrie et al.; 2015; USA | RCT; interaction (1 IG; 2 CG) | n=182; n CG active= 61, n CG passive=59;  age=11-14y; ♂=46% | objective (accelerometer), MVPA (weekly average duration; min/day) | 6 weeks; permanent; n.a. (theoretical background) | IG   - Reward system; gift card per week during course of the study for a realistic change in PA - Accelerometer use open automatically a website (feedback regarding user’s PA history, information about user’s current status within reward system)   CG 1   - Passive control (no intervention)   CG 1   - Active control (Dance Dance Revolution active video game) | **No sig. intervention effects**  group*time; *p*=.605  sex*group*time; *p*<.001 (favoured ♂) | **Different effects in** ♂**/**♀**in favour of** ♂  sex*group*time; *p*<.001 (favoured ♂) |
| **n.a.**  Haerens et al.; 2007; Belgium (a) | cluster, RCT; interaction (2 IG, 1 CG) | n=2434; n CG=735;  age= 13.1± 0.8y; ♂=62% | subjective (questionnaire) and objective (accelerometer); total PA (min/day; questionnaire), LPA, MVPA (min/day; accelerometer), meeting PA guidelines (%; accelerometer) | 9 months; permanent (school environment change), afternoon (sport materials), one parent meeting on PA; n.a. (theoretical background) | IG 1   - More opportunities to be physically active during school breaks, at noon or after-school hours - Extra PA on after-school hours and at noon - Extra sport materials (box) - Personal PA advice   IG2   - IG 1 + Parents interactive group meeting on PA and relationship with health   CG   - no intervention | **Sig. intervention effects**  total PA; post-hoc analyses: n.a.  LPA; time effects; IG1:CG; *p*=.08  LPA; time effects; IG2:CG; *p*<.05  MVPA; time effects; IG1:CG; *p*<.05  MVPA; time effects; IG2:CG; *p*<.05  meeting PA guidelines; time effects; post-hoc analyses: n.a. | **Same/similar intervention effects for both** ♂ **and** ♀  total PA; group*gender; n.s (*p=*n.a.)  LPA; group*gender; n.s (*p=*n.a.)  MVPA; group*gender; n.s (*p=*n.a.)  meeting PA guidelines; group*gender; n.s (*p=*n.a.) |
| **n.a.**  Haerens et al.; 2007; Belgium (b) | cluster RCT; interaction | n=231; n CG=142;  age= 13.2± 0.5y (CG), 13.2±0.8y (IG); ♂=52% | subjective (questionnaire); total PA (min/day) | 3 months; computer recommendations during class; n.a. (theoretical background) | IG   - Computer-tailored intervention (CD) with personal PA advice should encourage students to increase PA (for students who did not meet PA guidelines) or keep up with their PA habits   CG   - No intervention | **No sig. intervention effects**  time*group; n.s. (*p*=n.a.) | **Same/similar intervention effects for both** ♂ **and** ♀  time*group*gender; n.s. (*p*=n.a.) |
| **Fit´n`Fun Dudes**  Hardman et al.; 2009; UK | RCT; single sex/gender (♀) | n=29; n CG=15;  age= 10.7± 0.7y (CG), 10.5±0.9y (IG);  only ♀ | objective (pedometer); steps (mean steps per day) | 8 days; daily; n.a. (theoretical background) | IG:   - Children were given a personally-addressed letter from the Fit ‘n’ Fun Dudes (group of fictional, physically active peers designed to serve as a role model), pedometer step target with reward if reached (8 rewards in total, 1 for each day of intervention) - Parents were instructed to administer verbal praise when target was achieved   CG:   - No intervention | **No sig. intervention effects**  baseline; IG:CG; *p*=0.86  during intervention; IG:CG; *p*=0.03  follow-up; IG:CG; *p=.*81 | **No sig. intervention effects in** ♀ |
| **n.a.**  Hovell et al.; 2009; USA | RCT; dis. | n=117; n CG=49;  age= 10.7± 0.7y (CG), 10.5±0.9y (IG); ♂=46% | subjective (24-h PA recall); VPA (min/day; % of participants with ≥10min/day) | 8 weeks; weekly 90min for children and parents; n.a. (theoretical background) | IG   - Children were taught how to engage in bone-loading PA - Parents were taught behaviour management techniques to modify children’s behaviours   CG   - Procedures were identical to that of intervention group with topical information relating to child safety | **No sig. intervention effects**  VPA (min/day); time*group; n.s. for ♂ and ♀ (*p*=n.a.)  VPA (% of ≥10min/day); n.s. group differences at any point of measurement) | **Same/similar intervention effects for both** ♂ **and** ♀  Intervention effects were not sig. for ♂ and ♀ |
| **HEALTHY intervention**  Jago et al.; 2011; UK | cluster RCT; dis. | n=4063; n CG= 2003;  age=11.3± 0.6y (CG), 11.2±0.5y (IG); ♂=48% | subjective (questionnaire); MVPA (min/day) | 2.5 years; weekly sessions; n.a. (theoretical background) | IG   - Program of peer-led, teacher-facilitated learning activities known as FLASH modules (Fun Learning Activities for Student Health) - Modules contained sessions designed to foster self-awareness, knowledge, decision-making skills, peer involvement for health behaviour change - Social marketing campaign (e.g., encouraging PA) - More active PE curriculum   CG   - Activities were limited to recruitment and data collection only | **No sig. intervention effects**  time*group; *p*=.252  ♂; time*group; *p*=.211  ♀; time*group; *p*=.495 | **Same/similar intervention effects for both** ♂ **and** ♀  Intervention effects were not sig. for ♂ and ♀. |
| **Action 3:30**  Jago et al.; 2014; UK | cluster RCT; dis. | n=310; n CG=157;  age= 10.0± 0.05y; ♂=60% | objective (accelerometer); total PA (cpm), MVPA (min/day) | 20 weeks; one teaching of assistants, twice a week a 60min (Action 3:30 club), permanent equipment; SDT | IG   - Teaching assistants with training programme that focusses on delivering PA programme in after-school environment - Action 3:30 club with detailed session plans - Equipment for each intervention school to provide additional resources for Action 3:30 club   CG   - Only provided data, no other intervention | **Sig. intervention effects (only for total PA)**  Between group differences compared to IG and adjusted for baseline outcome value; results for follow-up  MVPA; adjusted difference in means=0.69 [95%CI=−3.4 to 4.8]  total PA; adjusted difference in means=6.0 [95%CI=−18.8 to 30.9] | **Same/similar intervention effects for both** ♂ **and** ♀  ♀; MVPA; adjusted difference in means=0.24 [95%CI=−5.3 to 5.8]  ♀; total PA; adjusted difference in means=2.6 [95%CI=2.1 to 20.1]  ♂; MVPA; adjusted difference in means=−0.59 [95%CI=−8.5 to 7.3]  ♂; total PA; adjusted difference in means=7.5 [95%CI=−50.4 to 65.5] |
| **‘Let Us Protect Our Future’**  Jemmott et al.; 2011; South Africa | cluster RCT; tested | n=1057; n CG=562;  age=9-18y; ♂=47% | meeting PA guidelines (% of participants with 2 days of strength-building activity and 4 days with 20min VPA or 5 days with 30min MPA) | 1 week; 1 hour per module, 2 modules on one day; TPB | IG   - Modules with interactive exercise, comic workbooks, home assignments to involve parents, exercise to upbeat music and examine the exercise on their heart by monitoring their pulse before and after exercise - Modules included attitudes, self-efficacy, and skills to practice healthful behavior   CG   - Intervention to practice abstinence and to use condoms | **Sig. intervention effects**  meeting PA guidelines; group effects (adjusted for baseline values); OR=1.56, *p*<.0001 (favoured IG) | **Same/similar intervention effects for both** ♂ **and** ♀  There were no intervention effects that were modified by gender. |
| **Mempis Girls’ health Enrichment Multi-site Studies (GEMS)**  Klesges et al.; 2010; USA | CT; single sex/gender (♀) | n=303; n CG=150;  age=9.3± 0.9y;  only ♀ | objective (accelerometer); total PA (cpm), MVPA (min/day) | 2 years, weekly (meetings), monthly (field trips), no information provided about theoretical background | IG   - Meetings and field trips with the community to provide interactive learning experiences in accordance to intervention goals - Focus on building awareness and community responsibility - Providing information, facilitating goal setting through demonstration and practical experience   CG   - Provide meaningful benefits with the goal of improving self-esteem and social efficacy; no focus on changing behaviours | **No sig. intervention effects**  Between group differences indicated treatment effects; adjusted for baseline values; 2y follow-up  total PA; adjusted difference in means=7.5 (95%CI=-14 to 21); n.s. (*p*=n.a.)  MVPA; adjusted difference in means=0.6 (95%CI=-1.3-2.4); n.s. (*p*=n.a.) | **No sig. intervention effects in** ♀ |
| **Komm mit ins gesunde Boot**  Kobel et al.; 2014; Germany | cluster RCT; interaction | n=1736; n CG=782;  age=7.06± 0.63y; ♂=51% | subjective (questionnaire); MVPA (% of participants with ≥4 days/week ≥60 min/day) | 12 months; permanent intervention; SCT | IG   - Materials offering action alternatives for recreational activities (without screen media) - PA integrated in primary school curriculum   CG   - Regular school curriculum | **No sig. intervention effects**  time*group; *p*=.191 | **Same/similar intervention effects for both** ♂ **and** ♀  time*group* gender; n.s.; *p*=n.a. |
| **"Le Sport Ca Me Dit" ("Sport is fun")**  Lanckriet et al.; 2017; France | CT; tested | n=180; n CG=107;  age=9.97± 0.86y (IG), 9.94± 0.83(CG); ♂=48% | objective (accelerometer); LPA, MPA, VPA, total PA (min/day) | 17 weeks; 2 times per week a 45min; n.a. (theoretical background) | IG   - Action groups with two recreation sessions of 45min per week using kit (consists of a box contains the equipment needed to play)   CG   - Did not have access to the kit | **No sig. intervention effects**  period 1: post-test; period 2: follow-up (follow-up was used for intervention effects)  total; period 1; IG:CG; *p*=.011  total; period 2; IG:CG; *p*=.074  LPA: period 1; IG:CG; *p*=.014  LPA; period 2; IG:CG; *p*=.057  MPA; period 1; IG:CG; *p*=0.532  MPA; period 2; IG:CG; *p*=.137  VPA; period 1; IG:CG; *p*=0.532  VPA; period 2; IG:CG; *p*=.137 | **Same/similar intervention effects for both** ♂ **and** ♀  Intervention effects on PA was not associated with gender. |
| **InPact study (part of the)**  Laukkannen et al.; 2015; Finland | cluster RCT; interaction | n=91; n CG=45;  age=6.07± 1.12y (IG), 6.20±1.13y (CG); ♂=46% | objective (accelerometer); MVPA (%/day) | 12 months; n.a. (frequency); SCT, TPB | IG   - Provide instructions (outdoor PA, PA with peers) - Provide information on consequences (lecture, information about how PA enhances health) - Prompting identification as a role model (information of concrete situations where parents act as physically active role model) - Provide general encouragement and progressive goal setting   CG   - Did not receive any counseling | **No sig. intervention effects**  group*time; *p*=.285 | **Same/similar intervention effects for both** ♂ **and** ♀  group*time*gender; *p*=.511 |
| **Active for Life Year 5 (AFLY)**  Lawlor et al.; 2016; UK | cluster RCT; dis. | n=2221; n CG=1157;  age=9.5± 0.3y; ♂=49% | objective (accelerometer); MVPA (min/day) | 1 school year; 16 lessons and 10 homework assignments; SCT | IG   - 16 lessons: 1 with 60min PA per day and 1 with reduced sedentary time - Homework assignments with parental involvement for activities at home   CG   - No intervention | **No sig. intervention effects**  ♀; IG:CG; OR=.05 (95%CI=-3.88 to 3.98); n.s (*p*=n.a.)  ♂; IG:CG; OR=-1.69 (95%CI=-6.34 to 2.97); n.s (*p*=n.a.) | **Same/similar intervention effects for both** ♂ **and** ♀  gender*group *time; *p*=.4 |
| **n.a.**  López-Fernández et al.; 2016; Spain | RCT; interaction (2 IG, 1 CG) | n=223; n CG=80;  age=7.1y; ♂=55% | objective (pedometer); steps/min | 2 months; daily 30min; n.a. (theoretical background) | IG1   - Six previous PE classes teaching games for recess - Playground markings and game equipment   IG1   - Playground markings and game equipment   CG   - No intervention | **Sig. intervention effects**  time*group; *p*<.001 (favoured IG1) | **Same/similar intervention effects for both** ♂ **and** ♀  time*group*gender; *p*=.118 |
| **Physical activity Leaders (PAL)**  Lubans et al.; 2011; Australia | RCT; single sex/gender (♂) | n=100; n CG=50;  age=14.2± 0.4y (CG), 14.4±0.7y (IG); only ♂ | objective; (pedometer); steps (per day) | 6 months; permanent to three times in one year; SCT | IG   - Enhanced school sport sessions - Interactive seminars - Lunch-time PA sessions; handbooks - Pedometers for self-monitoring - Parent newsletters - Tet messaging for social support   CG   - Wait-list control | **No sig. intervention effects**  time*group; *p*=.39 | **No sig. intervention effects in** ♂ |
| **Nutrition and Enjoyable Activity for Teen Girls (NEAT Girls)**  Lubans et al.; 2012; Australia | RCT; single sex/gender (♀) | n=357; n control=179;  age=13.2± 0.4y; only ♀ | objective (accelerometer); total PA (cpm), MVPA (min/day) | 12 months; permanent to three times in one year; SCT | IG   - Enhanced school sport sessions - Interactive seminars - Lunch-time PA sessions; handbooks - Pedometers for self-monitoring - Parent newsletters - Tet messaging for social support   CG   - Wait-list control | **No sig. intervention effects**  total PA; time*group; n.s. (*p*=n.a.)  MVPA; time*group; n.s. (*p*=n.a.) | **No sig. intervention effects in** ♀ |
| **Active Teen Leaders Avoiding Screen-time (ATLAS)**  Lubans et al.; 2016; Australia | cluster RCT; single sex/gender (♂) | n=361; n CG=180;  age=12.7± 0.5y; only ♂ | objective (accelerometer); total PA (cpm), MVPA (min/day) | 20 weeks; 20 PA sessions; SDT, SCT | IG   - Teacher professional learning workshops - Provision of fitness equipment - Researcher-led seminars for students - Face-to-face PA sessions delivered by teachers during PE - Lunch-time PA leadership sessions run by students - Pedometers for PA self-monitoring - Parental strategies for reducing recreational screen-time - Web-bases smartphone application   CG   - Wait-list control | **No sig. intervention effects**  total PA; time*group; *p*=.715  MVPA; time*group; *p*=.805 | **No sig. intervention effects in** ♂ |
| **Cretan Health and Nutrition Education Program**  Manios et al.; 2006; Greece | CT; dis. | n=425; n CG=238;  age=6.3y; ♂=48% | subjective (interview); MVPA (min/week), | 6 years; once per week (PE classes); n.a. (theoretical background) | IG   - Comprised a theoretical (4-6 classroom material per year) and practical part (2h PE classes/week) - Practical part was fitness-oriented, focusing on non-competitive and recreational forms of exercise in MPA   CG   - Usual PE | **Sig. intervention effects**  MVPA; ♂; group*time; *p*=.001  MVPA; ♀; group*time; *p*=*.*686 | **Different effects in** ♂**/**♀**in favour of** ♂  Sig. intervention effects only for ♂. |
| **n.a.**  McNeil et al.; 2009; Canada | cluster RCT; tested | n=360; n CG=120;  age=9.1± 0.7y; ♂=43% | subjective (questionnaire); overall PA (per day) | 1 school year; n.a. (frequency, theoretical background) | IG   - Focus on supporting families to help their children engage in more PA - Child’s interest in various activities was evaluated through a child-reported questionnaire - When programs of interest were not available, the connectors advocated for development or expansion of programs by volunteer groups and community agencies   CG   - No intervention | **No sig. intervention effects**  controlled for baseline values  IG:CG; *p*<.099  . | **Same/similar intervention effects for both** ♂ **and** ♀  Analyses revealed no significant gender differences |
| **Home- and childcare-based intervention to Promote Physical Activity (HIPPA)**  Mehtälä et al.; 2018; Finland | cluster RCT; dis. | n=69; n CG=;  age=3.25± 0.33y; ♂=48% | objective (accelerometer); MVPA, LPA, LMVPA (min/day), total PA (cpm/day) | 3 years; monthly letters and themes; SEM | IG   - Increase PA knowledge and skills of principals of childcare centres - Early Childhood Education and Care (ECEC) partnership (e.g. meetings, PA week calendar) - Facilities at childcare centres (e.g. more inspiring for PA to children; equipment available for children) - Outdoor playtime - Meetings with researchers to increase teachers’ knowledge - Increase Children’s motivation and self-efficacy   CG   - Continued normal care | **Sig. intervention effects (only for LPA and LMVPA)**  MVPA; baseline; IG:CG; *p*=.620  MVPA; post-test; IG:CG; *p*=.016  MVPA; follow-up; IG:CG; *p*=.174  LPA; baseline; IG:CG; *p*=.582  LPA; post-test; IG:CG; *p*=.016  LPA; follow-up; IG:CG; *p*=.012  LMVPA; baseline; IG:CG; *p*=.922  LMVPA; post-test; IG:CG; *p*=.030  LMVPA; follow-up; IG:CG; *p*=.016  total PA; baseline; IG:CG; *p*=.741  total PA; post-test; IG:CG; *p*=.107  total PA; follow-up; IG:CG; *p*=.066 | **Different effects in** ♂**/**♀**in favour of** ♀ **(MVPA, LMVPA) or in favour of** ♂ **(total PA, meeting PA guidelines)**  MVPA; ♀; group*time; *p*=.035  MVPA; ♂; group*time; *p*=.196  LMVPA; ♀; group*time; *p*=.035  LMVPA; ♂; group*time; *p*=.399  LPA; ♀; group*time; *p*=.062  LPA; ♂; group*time; *p*=.548  total PA; ♀; group*time; *p*=.0559  total PA; ♂; group*time; *p*=.791 |
| **n.a.**  Meier et al.; 2007; USA | RCT; dis. | n=255; n CG=134;  age=5-18y; ♂=66% | objective (pedometer) average steps (per day) | 6 weeks; permanent; n.a. (theoretical background) | Intervention   - Limited TV viewing (2 hours or 1 hour)   CG   - No intervention (unlimited TV viewing) | **No sig. intervention effects**  average steps; IG:CG; *p*=.023 (favoured CG)  average steps; group*gender; *p*=.053 | **Same/similar intervention effects for both** ♂ **and** ♀  Intervention effects were not sig. for ♂ and ♀. |
| **Kinder- und Jugendsport-studie**  Meyer et al.; 2014; Switzerland | cluster RCT; tested | n=502; n CG=205;  age=1st and 5th graders; ♂=53% | objective (accelerometer); total PA (cpm), MVPA (min/day) | 12 months; daily; SEM | Intervention   - Two additional PE lessons prepared by a team of expert PE teachers - 3 to 5 short activity breaks (2 to 5 min each) introduced every day during academic lessons; comprising motor skill tasks such as jumping or balancing on one leg - Daily PA homework of about 10 min   CG   - No intervention | **No sig. intervention effects**  adjusted for baseline values  total PA; IG:CG; *p*=.06  MVPA; IG:CG; *p*=.42 | **Same/similar intervention effects for both** ♂ **and** ♀  Secondary analyses that involved potential effect modifications by gender did not show any significant results. |
| **'Healthy Dads, Healthy Kids' (HDHK)**  Morgan et al.; 2011; Australia | RCT; tested | n=71; n CG=32;  age=8.2±2y; ♂=54% | objective (pedometer); steps (mean steps per day) | 3 months; 3 sessions during 3 months (with children); SCT, family systems theory | IG   - Fathers attending face-to-face group sessions; 3 with children - Program aims: help fathers to achieve their weight loss goals, become healthy role model, promote healthy behaviour in their children - Father/child sessions: Fundamental movement skills, rough and tumble play, health-related fitness, fun and active games   CG   - Wait-list control group; no information about intervention | **Sig. intervention effects**  steps; group*time; *p*<.001 (Cohen’s d=.74) | **Same/similar intervention effects for both** ♂ **and** ♀  No sex/gender differences displayed. |
| **“Dads And Daughters Exercising and Empowered”**  **(DADEE)**  Morgan et al.; 2018; Australia | RCT; single sex/gender (♀) | n=152; n CG=79;  age=7.7y;  only ♀ | objective (pedometer); steps (mean steps per day) | 8 weeks; weekly intervention; SCT, SDT | IG   - Combined education session: Father-only education session, daughter-only education session - Combined practical session - Sport skills programm; sports equipment pack; DADEE app   CG   - Wait-list control | **Sig. intervention effects**  baseline; IG:CG; n.s. (*p*=n.a.)  2 months; IG:CG; *p*=.02  9 months; IG:CG; *p*=.03 | **Sig. intervention effects in** ♀ |
| **Great activity programme**  Morris et al.; 2013; UK | CT; interaction | n=378; n CG=175;  age=9.75 ± 0.82 y; ♂=51% | objective (accelerometer and pedometer); steps (per day), | 7 months; 3 weekdays and 1 weekend day intervention; SCT, sport education instructional model | IG   - 3 highlight events (dance festival, walking event, running event) - interactive website for pupils, teachers and parents - Vacation activity planners   CG   - Usual PE | **Sig. intervention effects**  steps; group*time; *p*<.05  MVPA; group*time; *p*<.05 | **Different effects in** ♂**/**♀**in favour of** ♂ **(for MVPA)**  steps; group*time*sex; n.s. (*p*=n.a.)  MVPA; group*time*sex; *p*<.05 (favoured ♂) |
| **Sigue La Huella Intervention**  Murillo Pardo et al.; 2016; Spain | CT; interaction | n=682; n CG=314;  age=12-15y; ♂=54% | objective (accelerometer); MVPA (min/day) | 3 years; weekly intervention; SDT | IG   - Curricular channel: action plan which classroom teachers received training gin PA promotion and, in turn, involves students in the design of PA-related activities and inactivities - Non-curricular channel: regular information dissemination activities through which school members, families, community were kept updated about intervention and participation in institutional programs and special PA events in the community   CG   - No intervention | **Sig. intervention effects**  MVPA; group*time; *p*<0.001 | **Same/similar intervention effects for both** ♂ **and** ♀  MVPA; gender*time; *p=*.001  Group-by-gender interaction was not considered. |
| **n.a.**  Ni Mhurchu et al.; 2008; New Zealand | RCT; tested | n=20; n control=10;  age=12±1.5y; ♂=40% | objective (accelerometer) and subjective (questionnaire); total PA (cpm, PAQ-score)m LPA (min/day), MPA (min/day accelerometer and self-report), VPA (min/day accelerometer and self-report) | 12 weeks; n.a. (frequency, theoretical background) | IG   - Active video games; substitute usual non active video game play with active video games   CG   - Active game upgrade package upon competion of the study | **No sig. intervention effects**  results for 12 week follow-up, adjusted for baseline values  total PA; group differences; *p*=.6  No significant effect in time spent in MPA, VPA or mean PAQ-C scores. | **Same/similar intervention effects for both** ♂ **and** ♀  Intervention effects did not differ by sex. |
| **The Healthy School Start Study**  Nyberg et al.; 2015; Sweden | cluster RCT; tested | n=241; n CG=112; age=6.2± 0.3y; ♂=51% | objective (accelerometer); total PA (cpm), MVPA (min/day) | 6 months; 10x30min classroom activities; SCT | IG   - Health information brochures for parents - Motivational interviewing - Classroom activities (target children’s knowledge, attitudes and preferences and parent’s role modelling)   CG   - Waitlist control; continue usual practice | **No sig. intervention effects**  Results for follow-up; adjusted for baseline values  total PA; group effects; *p*=.51  MVPA; group effects; *p*=.60 | **Same/similar intervention effects for both** ♂ **and** ♀  sex*group; MVPA and total PA; n.s. (*p*=n.a.) |
| **n.a.**  Nyberg et al.; 2016; Sweden | cluster RCT; tested | n=378, n CG=196; age=6.3± 0.3y; ♂=50% | objective (accelerometer); total PA (cpm), MVPA (min/day) | 8 months; 10x30min classroom activities; SCT | IG   - Health information brochures for parents - Motivational interviewing - Classroom activities (target children’s knowledge, attitudes and preferences and parent’s role modelling)   CG   - Classes did not conduct any other organises healthy lifestyle activities | **No sig. intervention effects**  results for follow-up; adjusted for baseline values  total PA; group effects; *p*=.13  MVPA; group effects; *p*=.19 | **Same/similar intervention effects for both** ♂ **and** ♀  MVPA and total PA; group*sex; n.s. (*p*=n.a.) |
| **n.a.**  O'Dwyer et al.; 2012; UK | cluster RCT; tested | n=79; n CG=45; age=3.8± 0.6y; ♂=51% | objective (accelerometer); total PA (min/day) | 2.5 months; once every 2 weeks (70min); SEM | IG   - 70min sessions (10min registration and checking home activity completion, 60min delivery time) - Children: active play, parents: educational workshop (first 20 of 60min) - Active play (parents and children together; 40min) - Log book   CG   - Continue usual practice | **Sig. intervention effects**  Results adjusted for baseline values, reference category was CG  total PA; group effects; *ß*=4.70 (SE=.89; 95%CI=2.96-9.44); *p*<.05 | **Same/similar intervention effects for both** ♂ **and** ♀  Child’s sex was no significant predictor of total physical activity. |
| **Girls in Sport**  Okely et al.; 2017; Australia | cluster RCT; single sex/gender (♀) | n=1199; n CG=633;  age=13.6± 0.02y;  only ♀ | total PA, LPA, MPA, VPA, MVPA (min/da and % of day) | 18 months; once per month (meetings); Health promoting schools framework, Action learning approach | IG   - Formula curriculum: enhance school sports by trying to engage girls for at least 50% of class rime - School environment: e.g. after-school activity programs, modifying school policies related to use of equipment - Links with community: promotion out of school activities (e.g. use local facilities – fitness centres) - Meetings with a member of the research team - Funding and professional development activities (2-day training program and 2-day research symposium)   CG   - Continue usual practice | **No sig. intervention effects**  total PA; group*time; *p*=.894  LPA (min/day); group*time; *p*=.510  LPA (%); group*time; *p*=.554  MPA (min/day); group*time; *p*=.839  MPA (%); group*time; *p*=.890  VPA (min/day); group*time; *p*=.713  VPA (%); group*time; *p*=.848  MVPA (min/day); group*time; *p*=.848  MVPA (%); group*time; *p*=.850 | **No sig. intervention effects in** ♀ |
| **Sigue la Huella intervention (Follow the Footstep)**  Pardo et al.; 2014 | CT; dis. | n=682; n CG=314;  age=12-15y; ♂=54% | objective (accelerometer);MVPA (min/day) | 3 years; 1 classroom action plan per week; SEM, SDT | IG   - Curricular channel: Weekly classroom tutorial action plan in the classroom and PA education classes though teachers - Extra-curricular channel: Information dissemination activities for the broader community and participation in programs and special events   CG   - Continue usual practice | **Sig. intervention effects**  group*time; *p*<.001 | **Same/similar intervention effects for both** ♂ **and** ♀  group*time*gender; *p*=.0.54 |
| **LEAP (Lifestyle Education for Activity Program)**  Pate et al.; 2005; USA | cluster RCT; single sex/gender (♀) | n=2744; n CG=1221;  age=13.6y;  only ♀ | MVPA (% of participants with more than 2 blocks of 30min per day), VPA (% of participants with more than 1 block of 30min per day) | 3 years; permanent intervention; SEM | IG   - Instruction channel: activities that girls and young women typically enjoy were offered in addition to competitive sports and other traditional PE activities - Environment channel: role modelling by faculty and staff, increased communication about PA, promotion of PA by school nurse, family-based activities   CG   - No intervention | **Sig. intervention effects (only for VPA)**  MVPA; group*time; *p*=.53  VPA; group*time; *p*=.05 (favoured IG) | **Sig. intervention effects in** ♀ |
| **PACE+ for Adolescents (Patient-centered Assessment and Counseling for Exercise + Nutrition)**  Patrick et al.; 2006; USA | RCT; dis. | n=819; n CG=395;  age=2.7± 1.3y; ♂=50% | MVPA (min/week), | 12 months; one computer-based expert assessment, 11 telephone counselling calls (10-15min); behavioural determinants model, SCT, trans-theoretical model of behaviour change | IG   - Computer-supported intervention; coupled with a printed manual to take home - Parent intervention intended to help parents encourage behavior change attempts - 16-section printed Teen Guide, mail, and telephone support changes in target behaviour   CG   - Adaption of the SunSmart sun protection behaviour programme | **No sig. intervention effects**  MVPA; ♀; time*group; *p*=.90  MVPA; ♂; time*group; *p*=.17 | **Same/similar intervention effects for both** ♂ **and** ♀  Intervention effects were sig. for ♂ and ♀. |
| **Salud Integral (=Compensive Health), SI!**  Peñalvo et al.; 2013; Spain | RCT; tested | n=2062; n CG=920;  age=3-5y; ♂=59% | subjective (questionnaire); PA habits (score) | 12 month; permanent intervention; SCT, trans-theoretical models in health promotion | IG   - Classroom materials (e.g. different resources such as healthy tales, educational games) - Take-home activities - Cards to address managing emotions (10 hours per academic year) - “Healthy tip” (i.e. healthy activity for family to share over weekend) - Teachers: intervention website (download all resources)   CG   - Information about the content of intervention for children but not aware of the specifics or main objectives of intervention - Follow regular curriculum | **Sig. intervention effects**  adjusted for baseline scores  follow-up; IG:CG; *p<*.001 | **Same/similar intervention effects for both** ♂ **and** ♀  Sex-by-group interaction was not significant. |
| **PhysicaL Activity in Youth (PLAY),**  Pienaar et al.; 2012; South Africa | cluster CT; dis. | n=369; n CG=87;  age=14.9±1.4y (♂), 14.3±1.4y (♀); ♂=40% | subjective (recall questionnaire); total PA (min/day) | 10.5 months; 2-3 times per week (60min each); n.a. (theoretical background) | IG:   - 20min aerobic exercise at moderate to high intensity to the beat music, 20min of active participation in sports related to ball games/skills, 20min strength and flexibility exercise   CG:   - Continue usual practice | **No sig. intervention effects**  group*time; *p*=.60 | **Different effects in** ♂**/**♀**in favour of** ♂  group*time*gender; *p*=.07  ♂; group*time; *p*=.004  ♀; group*time; *p*=.42 |
| **No name reported**  Razak; 2018; Australia | cluster RCT; dis. | n=439; n control=;  age= 3.73 ±0.59y; ♂=53% | objective (accelerometer); MVPA (min/day) | 3 months; 3 times a 15min (outdoor play), 4 times (support intervention staff), permanent (materials); no information provided about intervention content | Intervention   - Outdoor free-play periods - Support by the intervention staff (telephone calls and visits) - Materials for services: national guidelines; “Get up & Grow” materials, benefits of outdoor play resources   Control  Usual practice | **Sig. intervention effects**  ♂ Baseline  IG=64.69 (21.03)  CG= 57.20 (17.85)  ♂Follow Up  IG=64.85 (19.49)  CG=57.13 (17.05)  ♀ Baseline  IG=50.30 (18.56)  CG= 46.08 (14.99)  ♀ Follow Up  IG=50.27 (17.88)  CG=47.11 (15.00) | **Same/similar intervention effects for both** ♂ **and** ♀  Group x subgroup (reference category is ♀)  estimate (95% CI)=2.87 (-4.69-10.43)  p=0.41 |
| **n.a.**  Reilly et al.; 2006; UK | RCT; dis. | n=545; n CG=277;  age=4.1±03y; ♂=50% | objective (accelerometer);total PA (cpm) | 6 months; 3 times per week (30min session); permanent (information for home); n.a. (theoretical background) | IG   - Nursery elements: 30min sessions of PA - Home elements: family resource packs with information material, health education leaflets, posters focusing on increasing PA through walking and playing   CG   - Continue usual practice | **No sig. intervention effects**  6 months; group*time; *p*=.87  12 months; group*time; *p*=.90 | **Same/similar intervention effects for both** ♂ **and** ♀  controlled for baseline value  sex*group; n.s. (*p*=n.a.) |
| **Girls on the Move (GOTM)**  Robbins et al.; 2012; USA | cluster RCT; single sex/gender (♀) | n=69; n CG=20;  age=11.49± 0.84y (IG), 11.44±0.67y (CG);  only ♀ | objective (accelerometer)MVPA (min/h) | 6 months; daily (after-school PA club), 3 x 20min (sessions); HPM | IG   - After-school PA club - Face-to-face motivational, individually tailored counseling session with registered nurse during the school day   CG   - 90min after-school workshops once a month for 6months with no topics of PA | **No sig. intervention effects**  analyses controlled for baseline measure  CG:IG; *p=*.35 | **No sig. intervention effects in** ♀ |
| **Girls on the Move (GOTM)**  Robbins et al.; 2018; USA | cluster RCT; single sex/gender (♀) | n=1543; n CG=766;  age=12.05 ±1y;  only ♀ | objective (accelerometer)MVPA (min/h) | 17 weeks; 3 times per week (PA club); 2 sessions a 15-20min (face-to-face session, once (internet session); Health Promotion Model, SDT | IG   - After-school PA club (conducted by club manager and instructors) - Face-to-face motivational individually tailored counselling sessions - Interactive internet-based session   CG   - Usual school offerings | **No sig. intervention effects**  post intervention (results controlled for baseline values)  MVPA; IG:CG; n.s. (*p*=n.a.)  follow-up (results controlled for baseline values)  MVPA; IG:CG; n.s. (*p*=n.a.) | **No sig. intervention effects in** ♀ |
| **SNAP (Scouting Nutrition & Activity Program)**  Rosenkranz et al.; 2010; USA | cluster RCT; single sex/gender (♀) | n=72; n CG=39;  age=10.5y;  only ♀ | objective (accelerometer);MVPA (days per week of 60min MVPA) | 7 months; 8 times a 60-90min (educational curriculum), once (troop leader training); SCT | IG   - Educational curriculum (e.g. target behaviors, worksheet for goal setting and self-monitoring, PA recreation session etc.) - Troop leader training - Support by intervention staff (e-mail and phone support)   CG   - Usual practice | **No sig. intervention effects**  time*group; *p*>.05 | **No sig. intervention effects in** ♀ |
| **n.a.**  Rostami-Moez et al.; 2017; Iran | cluster RCT; single sex/gender (♀) | n=314; n CG=165;  age=8-11y;  only ♀ | subjective (questionnaire); total PA (days/week) | 8 month; once per week (PA training), daily (log book), permanent (newspapers, banner, handcrafts), 2 times (education sessions for teachers); trans-theoretical model | IG   - Training in the field of PA; importance and benefits of adequate PA - Friendship teams to walk to school - Wall newspapers and handcrafts for PA - Workout CD and music; reminder bracelets, brochures - Education sessions for teachers, school staffs and students’ parents - PA related banner with content of 60min MVPA   CG   - Usual practice; after completion of study received educational materials and one session of education | **Sig. intervention effects**  time*group; *p*=.001 | **Sig. intervention effects in** ♀ |
| **n.a.**  Salminem et al.; 2005; Finland | CT; dis. (different outcomes for ♂ for and ♀); (1 IG; 2 CG) | n=1055; n CG1=200, n CG2 2= 423;  age=8-14y; ♂=52% | subjective (questionnaire); for ♀: exertion of exercise; for ♂: frequency of exercise | 33 months; permanent (reading materials), 5 times (counselling sessions); health education approach | IG   - Reading materials provided by voluntary organizations (e.g. effects of nutrition, exercise) - Counselling sessions: 2 for children at school and 3 for children and their family members at home (e.g. risk factors of diseases and PA)   CG   - Usual practice; regular health service options available to anyone - CG 1 with history of cardio-vascular disease, CG 2 group two without | **Sig. intervention effects**  exertion; ♀; time*group; *p*=.030 (favoured IG)  frequency; ♂; time*group; *p*=.001 (favoured IG) | **Same/similar intervention effects for both** ♂ **and** ♀  Intervention effects were sig. for ♂ and ♀ (different outcomes). |
| **Switch Plax**  Salmon et al.; 2008; Australia | RCT; dis. (different outcomes ♂ for and ♀); (3 IG, 1 CG) | n=306; n CG=62;  age=10.8y; ♂=48% | objective (accelerometer); total PA (counts per day), VPA (min/day; ♂), MPA (min/day; ♀) | 6 months; 19 lessons a 40-50min; SCT | IG 1   - Behavioural modification (e.g. Increasing PA, the home environment, community environment, intelligent viewing and decreasing sedentary behaviour)   IG2   - Intervention 2: Fundamental movement kills (e.g. dodge and kick, vertical jump, run and throw)   IG3   - Intervention 3: Behavioural modification and fundamental movement skills   CG   - Continue usual practice | **Sig. intervention effects**  controlled for baseline values  VPA; ♂; IG1:CG; *p*<.01  VPA; ♂; IG2:CG; *p*<.001  VPA; ♂: IG3:CG; n.s. (*p*=n.a.)  total PA; ♂; IG1:CG; *p*<.05  total PA; ♂; IG2:CG; *p*<.001  total PA; ♂: IG3:CG; n.s. (*p*=n.a.)  MPA; ♀; IG1:CG; *p*<.01  MPA; ♀; IG2:CG; n.s. (*p*=n.a.)  MPA; ♀: IG3:CG; n.s. (*p*=n.a.)  total PA; ♀; IG1:CG; *p*<.05  total PA; ♀; IG2:CG; n.s. (*p*=n.a.)  total PA; ♀: IG3:CG; n.s. (*p*=n.a.) | **Different effects in** ♂**/**♀**in favour of** ♂  Total PA only sig for ♂.  VPA was measured only for ♂ and MPA was measured only for ♀ |
| **Swtich-2-Activity**  Salmon et al.; 2010; Australia | RCT; tested | n=957; n CG=490; age=10.3±0.62y; ♂=42.0% | subjective (self-reported survey); MVPA (min/day, min/weekday) | 7 weeks; weekly; SCT | IG   - Behaviour change strategies: self-monitoring, behavioural contracting and budgeting of television viewing - Lessons: introduction of PA and heath, patterns of TV viewing, selective TV viewing, decision-making skills, identifying alternative strategies, Switch-2-Activity games and pedometer   CG   - Wait-list control group | **No sig. intervention effects**  time*group; n.s. (*p*=n.a.)  Interactions by sex were not significant. | **Same/similar intervention effects for both** ♂ **and** ♀  Interactions by sex were not significant |
| **n.a.**  Sanaeinasab et al.; 2012; Iran | cluster RCT; dis. | n=165; n CG=85;  age=13.99± 0.4y; ♂=53% | subjective (questionnaire)  total PA (min/week) | 2 months; 10 sessions (1-1.15hrs interactive sessions), once (family workshop), HPM, trans-theoretical model | IG   - Interactive sessions; attempted to see the viewpoints of participants about personal and environmental obstacles toward PA by performing interpersonal consulting or group discussion - Family workshop (e.g. program goals) - Exercise program (2 football and footrace competitions)   CG   - Regular school programs | **Sig. intervention effects**  baseline; IG:CG; *p*=.238  follow-up; IG:CG; *p*<.001 (favoured IG) | **Different effects in** ♂**/**♀**in favour of** ♂  gender*group (controlled for baseline values); *p*<.001 (favoured ♂); Intervention effects for both (♂ and ♀) |
| **n.a.**  Schneider et al.; 2011; USA | CT; single sex/gender (♀) | n=122; n CG=59;  age=15.04 ±.8y;  only ♀ | subjective (3-day PA recall); VPA, MVPA (min/day) | 3 years; daily for 60min; n.a. (theoretical background) | IG   - Educational discussion to the health benefits of exercise and strategies for adopting and active lifestyle - Supervised activities were selected based on student input (e.g. weightlifting, yoga)   CG   - No instructions with regard to PA | **Sig. intervention effects (only for MVPA)**  MVPA; time*group; *p*<.05  VPA; time*group; n.s. (*p*=n.a.) | **Sig. intervention effects in** ♀ |
| **n.a.**  Schneider et al.; 2008; USA | CT; single sex/gender (♀) | n=120; n CG=67;  age=15.04 ±0.8y;  only ♀ | subjective (3-day PA recall); VPA (% of participants doing some VPA) | 9 months; daily; hedonic theory, SDT | IG   - Educational discussion to the health benefits of exercise and strategies for adopting and active lifestyle - Elements of intervention were included to make the class appealing for low active females (e.g. participants had input into the choice of actives)   CG   - No instructions with regard to PA | **Sig. intervention effects**  time*group; *p=*.001 | **Sig. intervention effects in** ♀ |
| **n.a.**  Schofield et al.; 2005; Australia | CT; single sex/gender (♀) (2 IG; 1 CG) | n=85; n CG=30;  age=15.8± 0.8y;  only ♀ | objective (pedometer) and subjective (questionnaire); steps (objective, per 4 days), MVPA and VPA (subjective, number of 30min blocks for 3 days) | 12 weeks; 1 group meeting per week; n.a. (theoretical background) | IG   - Intervention 1= pedometer group (encouraged to increase their activity by daily average of 1-2000 steps for each week until they reached 10.000 steps per day) - Intervention 2= Minutes group (encouraged to add a daily average of 10-15min of activity for each week, until they reached a daily average of at least 30-60min/day)   CG   - No intervention | **Sig. intervention effect (only for steps)**  steps; time*group; *p*<.001 (favour IG)  MVPA; time*group; *p*=.94  VPA; time*group; *p*=.81 | **Sig. intervention effects in** ♀ |
| **Peer-Led physical Activity iNtervention (PlAN-A)**  Sebire et al.; 2018; UK | CT; single sex/gender (♀) | n=427; n CG=158;  age=12-13 years;  only ♀ | objective (accelerometer);  MVPA (min/day) | 2.5 months; 2 days peer-supporter training (6hours each day); Diffusion on Innovation theory; SDT | IG:   - Trainers with three-day train-the-trainers programme approximately one week before delivery - Peer-supporter training was provided for each school to the top of about 18 girls nominated to be a peer-supporter and delivered by two trainers; peer-supporter returned to school and informally diffused messages about PA to friends   CG:   - No intervention but pupils completed identical measurements to the intervention schools | **Sig. intervention effects**  controlled for baseline values  time 1; IG:CG; ß=1.11 (95%CI=-4.31 to 6.55); *p*=n.a.  time 2; IG:CG; ß=6-09 (95%CI)=1.43 to 10.76; *p*=n.a. | **Sig. intervention effects in** ♀ |
| **n.a.**  Sigmund et al.; 2012; Czech Republic | CT; dis. | n= 176; n CG=88; age=6-7 years; ♂=52% | objective (pedometer); steps (per day) | 24 months; daily (PA, breaks), 3-4 times per week (recess); n.a. (theoretical background) | IG:   - 20-min recess with PA content - PA (playing) undertaken during after-school nursey (up to 90min) - 2-3 short breaks per day; movement playing in classroom   CG:   - Continue usual practice | **Sig. intervention effects**  steps; time*group; *p*<.0001 | **Same/similar intervention effects for both** ♂ **and** ♀  time*group *gender; p=.6 |
| **Active Teen Leaders Avoiding Screen-time (ATLAS) intervention** Smith et al.; 2014; Australia | cluster RCT; single sex/gender (♂) | n=361; n CG= 180; age= 12.7±0.5y;  only ♂ | objective (accelerometer);total PA (cpm), MVPA (%/day) | 5 months; 2 times (workshops for teachers), weekly (90min, enhanced sport sessions), 6 times (20min, lunchtime PA sessions), permanent (smartphone app, pedometers); self-determination theory, SCT | IG:   - Teachers professional development (workshops) - Parental newsletters (information about potential consequences of excessive screen use among youth, strategies for reducing screen-based recreation in family home, tips for avoiding conflict when implementing rules) - Researchers-led seminars for students (seminars that provide information) - Enhanced school-sport sessions - Lunchtime PA mentoring sessions - Smartphone app and website (PA mentoring) - Pedometers   CG:   - Continue usual practice; will receive equipment pack and condensed version of the program after last assessment | **No sig. intervention effects**  total PA; time*group; *p*=.41  MVPA; time*group; *p*=.14 | **No sig. intervention effects in** ♂ |
| **n.a.**  Smpokos et al.; 2010; Greece | cluster RCT; tested | n= 798; n CG=439; age=7.6± 0.9y; ♂=51% | subjective (questionnaire); MVPA (min/week) | 12 months; n.a. (frequency, theoretical background) | IG   - Educational program was not described in detail   CG   - No intervention | **Sig. intervention effects**  controlled for baseline values  IG:CG; *p*=.006 | **Same/similar intervention effects for both** ♂ **and** ♀  The study indicates a favourable effect of the intervention program on physical activity level for both sexes at the end of the intervention programme. |
| **The Pathways Intervention**  Stevens et al.; 2003; USA | cluster RCT; dis. | n= 1477; n CG=688; age=8-11y; ♂=52% | subjective (PA recall); total PA (Met-time) | 3 years; n.a. (frequency), social learning theory | IG   - PE component which increased PA at school - Classroom curriculum focused on knowledge and practiced related to lifestyle habits - Family component aimed at involving parents of children in participating in program   CG   - No intervention | **Sig. intervention effects**  ♂; time*group; *p*=.022  ♀; time*group; *p*=.020 | **Same/similar intervention effects for both** ♂ **and** ♀  Intervention effects were sig. for ♂ and ♀. |
| **Girlsfriends for KEEPS (Keys to eating, Exercising, Playing, and Sharing)**  Story et al.; 2003; USA | RCT; single sex/gender (♀) | n= 54; n CG=28; age=9.3± 0.9y; only ♀ | objective; accelerometer; total PA (cpm, Met-time), MVPA (min/day) | 3 months; 2-3 times per week for 60min (after-school program); SCT | IG   - After-school Program: Club meetings (major component was increasing PA levels with variety of choice of activities) - Family involvement (e.g. take home packs, family night events)   CG   - Received program over 12 weeks unrelated to PA; arts and crafts, self-esteem activities, creating memory books, workshop on African percussion instruments | **No sig. intervention effects**  Post intervention effects; analyes adjusted for baseline values  total PA; group effects; *p*=.12  MVPA; group effects; *p*=.83 | **No sig. intervention effects in** ♀ |
| **Physical Acitivty 4 Eceryone (PA4E1)**  Sutherland et al.; 2016; Australia (a) | cluster RCT; dis. | n= 1150; n CG=505; age=12.0y; ♂=49% | objective (accelerometer);MVPA (min/day, %/day) | 24 months; once per week (10 week program during school); SCT | IG   - Training of PE teachers to maximizing MVPA during class time - Student PA plans within PE lessons - Enhance school program; 10-week program during school (focusing on lifelong PA) - Modification of school policies: aimed to enhance student PA - PA program during school breaks provided with equipment - Promotion of community PA providers - Parent engagement via newsletters and school website promoting PA   CG   - Continue usual practice | **Sig. intervention effects**  MVPA (min/day); ♂; time*group; *p*=.02  MVPA (%);♂; time*group; *p*=.02  MVPA (min/day); ♀; time*group; *p*=.05  MVPA (%);♀; time*group; *p*=.03 | **Same/similar intervention effects for both** ♂ **and** ♀  Intervention effects were sig. for ♂ and ♀. |
| **Physical Activity 4 Everyone (PA4E1)**  Sutherland et al.; 2016; Australia (b) | cluster RCT; dis. | n= 1150; n CG=505; age=12.0y; ♂=49% | objective (accelerometer); MVPA (min/day) | 24 months; once per week (10 week program during school); SCT | IG   - Training of PE teachers to maximizing MVPA during class time - Student PA plans within PE lessons - Enhance school program; 10-week program during school (focusing on lifelong PA) - Modification of school policies: aimed to enhance student PA - PA program during school breaks provided with equipment - Promotion of community PA providers - Parent engagement via newsletters and school website promoting PA   CG   - Continue usual practice | **Sig. intervention effects**  Results controlled for baseline values; reference group was CG  MVPA; group effects; adjusted difference in means=7.0mins (95%CI=2.7 to 11.4); *p*<.002  MVPA, ♂; mean difference in change=10.4mins (95%CI=1.7 to 10.7); *p*<.015  MVPA; ♀; mean difference in change=4.0min; 95%CI=0.1-6.5); *p<*0.5 | **Same/similar intervention effects for both** ♂ **and** ♀  Intervention effects were sig. for ♂ and ♀. |
| **The Learning, Cognition & Motion study (LCoMotion)**  Tarp et al.; 2016; Denmark | cluster RCT; tested | n= 632; n CG=438; age=12.9± 0.6y; ♂=49% | objective (accelerometer); total PA (cpm), MVPA (min/day) | 20 weeks; daily (PA during school and recess); n.a. (theoretical background) | IG   - PA activity during school (60min) - Teacher: course on incorporating PA into academic class - Scheduled PA during recess - PA homework (booklet) - Active transportation (cycling campaign) - Activity watch   CG   - Usual practice | **No sig. intervention effects**  controlled for baseline values  total PA; IG:CG; *p*=.77  MVPA; IG:CG; *p*=.64  No interaction between gender and school type was evident in any models. | **Same/similar intervention effects for both** ♂ **and** ♀  No interaction between gender and school type was evident in any models. |
| **n.a.**  Taymoori et al.; 2008; Iran | cluster RCT; single sex/gender (♀) (2 IG, 1 CG) | n= 161; n CG=52; age=14.79 ± 0.44y; only ♀ | subjective (PA recall); total PA (min/week); MVPA (min/day) | 6 months; 4 times during study period (group educational session and individual counselling); HPM, Trans-theoretical Model | IG   - Intervention group 1: based on Health Promotion Model - Intervention group 2: based on transtheoretical model - 60min group educational sessions (e.g. perceived benefits and barriers of PA); only for intervention group 2: counter conditioning and stimulus control)   CG   - Usual practice | **Sig. intervention effects**  MVPA; group*time; *p*<.005  total PA; group*time; *p*<.005 | **Sig. intervention effects in** ♀ |
| **The LOOK study**  Telford et al.; 2016; Australia | cluster RCT; dis. | n= 853; n CG=375; age=8.1± 0.3y; ♂=51% | objective (pedometer, accelerometer); steps (per day; % of ≥12.000 steps/day), MVPA (min/day, % of ≥60min/day) | 4 years; 2 times per week for 45min; guided discovery method of teaching | IG   - PE taught by a Bluearth specialist (university trained and qualified PE teaches with further specialised training in Bluearth approach); wihich amounted to 90min of the mandatory weekly PE   Control   - Usual practice | **No sign. intervention effects**  steps/day; ♂; group*time; *p*=.131  MVPA; ♂; group*time; *p*=.130  steps/day; ♀; group*time; *p*=.209  MVPA; ♀; group*time; *p*=.207 | **Same/similar intervention effects for both** ♂ **and** ♀  Intervention effects were not sig. for ♂ and ♀. |
| **SPACE (for physical activity)**  Toftager et al.; 2014; Denmark | cluster RCT; tested | n=797; n CG=421; age= 12.5±0.63y (IG), 12.5±0.61y (CG); ♂=50% | objective (accelerometer); total PA (cpm), MVPA (min/day) | 24 months; permanent; SEM | IG:   - Upgrade existing outdoor areas at the school for PA - Develop and build playgrounds - improve safety for active transport - Establish an after school fitness program - Formulate and implement school PA policy - Educate teachers as ‘‘kick-starters” - Establish school play patrol: older students were trained to initiate play and games for minors during school recess - Mandatory outdoor recess - School traffic patrol: older students helped minors cross the streets near the school - Educate and train students in safe cycling, - School project/theme week once a year focusing on learning about and doing PA during school lessons   CG   - No intervention | **No sign. intervention effects**  controlled for baseline values  total PA; IG:CG; *p*=.09  MVPA; IG:CG; *p*=.587 | **Same/similar intervention effects for both** ♂ **and** ♀  Stratification by sex resulted in no significant differences in intervention effects. |
| **n.a.**  Vašíčková et al.; 2013; Poland | cluster RCT; dis. | n=495; n CG=247  age=15.68± 0.79y (♀ IG), 15.63±0.84y (♂ IG), 15.97±0.92y (♀ CG), 15.66±0.83y (♂ CG); ♂=44% | subjective (questionnaire); total PA (Met-min/week) | 1 month; permanent; n.a. (theoretical background) | IG:   - Pedometer use - Motivational recording brochures (record tables for data from pedometer, possibility of graphic expressions of daily steps, record tables for movement / inactivity)   CG:   - No intervention | **No sign. intervention effects**  No change in total PA from T0 to T1 for ♀ and ♂ *p*=n.a.  T0; ♀: ♂; IG; *p*=.005 (favoured ♂)  T0; ♀: ♂; KG; *p*=.015 (favoured ♂)  post-test; ♀: ♂; IG; *p*=.631  post-test; ♀: ♂; KG; *p*=.002 | **Same/similar intervention effects for both** ♂ **and** ♀  binary logistic regression analyses  gender: OR=2.64; CI=1.33 to 4.39; *p*=.06 |
| **IDEFICS study (Identfication and prevention of Dietary- and lifestyle included health EFfects In Children and InfantS)**  Verbestel et al.; 2015; 8 European Countries | CT; dis. | n= 7413; n CG= 231; age=10.87± 0.82y; ♂=51% | objective (accelerometer);total PA (cpm), MVPA, LPA (%/day) | 24 months; 8 healthy weeks, permanent intervention; SEM | IG   - Community level: involvement of community partners, long term media campaign and public relation strategies, lobbying for community environmental and policy interventions - School level: building partnerships, education of children (8 healthy weeks), environmental changed related to PA- the active playground, health related PE curricula - Family level: education of parents (materials)   CG   - No intervention | **No sign. intervention effects**  total PA; ♂; time*group; *p*=.062  total PA; ♀; time*group; *p*=.339  LPA; ♂; time*group; *p*=.478  LPA; ♀; time*group; *p*=.139  MVPA; ♂; time*group; *p*=.070  MVPA; ♀; time*group; *p*=.221 | **Same/similar intervention effects for both** ♂ **and** ♀  Intervention effects were not sig. for ♂ and ♀. |
| **UP4FUN**  Verloigne et al.; 2012; Belgium | cluster RCT; dis. | n=372; n CG=231; age=10.9± 0.7y; ♂=44.2% | objective (accelerometer);LPA, MVPA (%/day) | 1.5 months; once per week (newsletter, lessons per week), once (family fun event, teacher training), permanent (materials, manual, motivation factors); Model of planned promotion for Population health; SEM | IG:   - 1 hour teacher training - Teacher manual (outline of each lesson; activity breaks), materials to be handed out on pupils - One specific theme every week: (1) introduction in the project, (2) awareness of sitting time, (3) evaluation of sitting time, (4) influencing factors at home, (5) possibilities for active breaks/transportation, (6) family fun event - Newsletter - Motivation factors: step counters, stickers   CG:   - Continue usual practice | **No sig. intervention effects**  LPA; ♂; time*group; n.s. (*p*=n.a.)  MVPA; ♂; time*group; n.s. (*p*=n.a.)  LPA; ♀; time*group; n.s. (*p*=n.a.)  MVPA; ♀; time*group; n.s. (*p*=n.a.) | **Same/similar intervention effects for both** ♂ **and** ♀  Intervention effects were not sig. for ♂ and ♀. |
| **Sports, Play, and Recreation for Youth (SPARK) program**  Verstraete et al.; 2007; Belgium | cluster RCT; tested | n=764 (16 schools); n CG=8 schools;  age= 11.2± 0.7y; ♂=49% | subjective (questionnaire); LPA, MPA, VPA, MVPA (min/day) | 17 months; daily (implementation of guidelines in PE lessons), 6 lessons (health education), permanent (equipment), once per week or less (extra-curricular PA); n.a. (theoretical background) | IG:   - Implementation of didactical guidelines in all PE lessons - Classroom-based health education lessons (promote knowledge and skills including goal-setting, time planning, problem-solving, self-talk) - Extracurricular PA promotion programme (game equipment provided during lunch and recess) and on voluntary basis   CG:   - No intervention | **Sig. intervention effects (for MPA and MVPA)**  LPA; time*group; n.s. (*p*=n.a.)  MPA; time*group; *p*<.01  VPA; time*group; n.s. (*p*=n.a.)  MVPA; time*group; *p*<.01  LPA; time*group; n.s. (*p*=n.a.) | **Same/similar intervention effects for both** ♂ **and** ♀  No significant gender differences were found on the accelerometer data. |
| **Health Legacy Project (HLP)**  Wang et al.; 2018; China | cluster RCT; tested | n= 9859; n CG=4583; age=9.0± 0.01y; ♂=53% | subjective (questionnaire); MVPA (min/week, percent of students with increased MPA time at study end) | 9 months; permanent intervention components; n.a. (theoretical background) | IG   - Classroom curricula (knowledge and skills of PA) - School environment support (posters and slogans to engage in sufficient PA) - Family involvement (Parents’ health class, parents’ homework) - Fun programs/events (composition writing with a focus on PA, painting class with the theme of PA events in daily life, team competition on knowledge and skills regarding obesity and PA)   CG   - Usual practice | **Sig. intervention effects (for % of students with MPA increase)**  adjusted for baseline PA  MVPA (min/week); IG:CG; *p*=.36  % of students with MPA increase; IG:CG; *p*<.01 | **Same/similar intervention effects for both** ♂ **and** ♀  Estimates did not suggest significant differences in intervention effects across gender. |
| **Healthy Eating and Activity Time**  Weaver et al.; 2017; USA | cluster RCT; dis. | n= 1830; n control=926; age=7.9± 2.0y; ♂=55% | objective (accelerometer); MVPA (min/day, % of meeting guidelines of 60min/day) | 12 months; daily; Theory of Expanded, and Extended, and Enhanced Opportunities | IG   - PA schedules; extend existing PA opportunities to three hours per day - Encourage to expand PA opportunities by integrating short activity breaks and exchange inactive field trips for more activity - Staff with professional development training covering the topic of enhancing the amount of activity in children (2 booster sessions)   CG   - Support for improving types of foods/ beverages served | **Sig. intervention effects**  % of meeting guidelines of 60min/day; ♂; group*time; *p*=.023  % of meeting guidelines of 60min/day; ♀; group*time; *p*<.001  MVPA (min/day); ♂; group*time; *p=*.036  MVPA (min/day); group*time; ♀; *p*=<.001 | **Same/similar intervention effects for both** ♂ **and** ♀  Intervention effects were sig. for ♂ and ♀. |

*Note*: CG=control group; CI=confidence interval; CT=controlled trial; dis.=disaggregated; HPM= Health Promotion Model; IG=intervention group; LPA=light physical activity; LMVPA=light-to-vigorous physical activity; n.a.= not available; n.s.=not significant; MPA=moderate physical activity; MVPA=moderate to vigorous physical activity; OR=odds ratio; PA=physical activity; PE=physical education; RCT=randomized controlled trial; SCT=social cognitive theory; SDT=self-determination theory; SEM=socio-ecological model; TPB=theory of planned behaviour; UK= United Kingdom; VPA=vigorous physical activity.
