# Supplementary figures and images for: A sex/gender perspective on interventions to promote children’s and adolescents’ overall physical activity: results from genEffects systematic review
Source: BMC Pediatr. 2020 Oct 10;20:473. doi: 10.1186/s12887-020-02370-9 (PMC7547493; doi:10.1186/s12887-020-02370-9)

**Additional file 4: Risk of bias summary table**


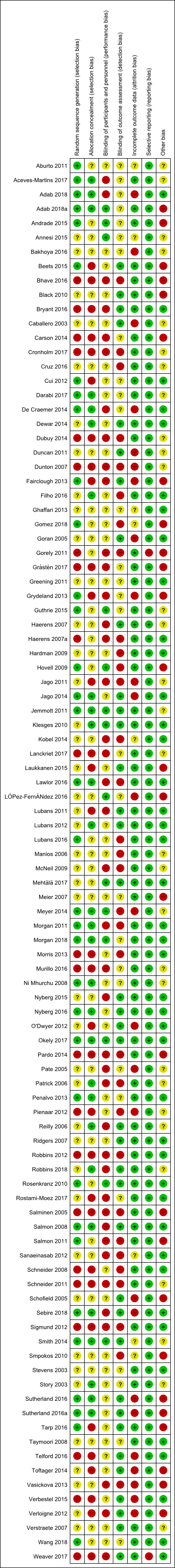


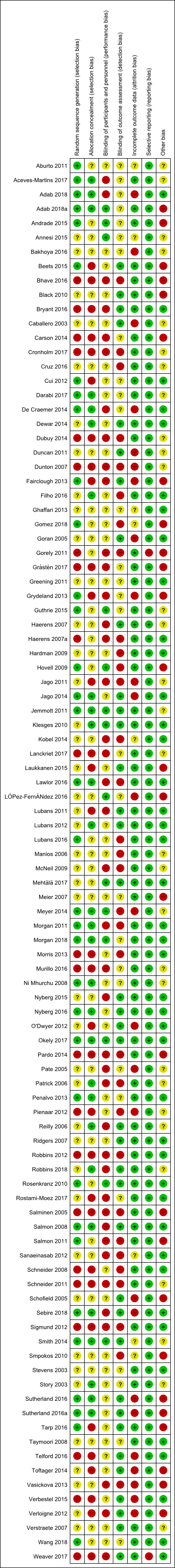

Supplement: Supplementary file 4 — Additional file 4. Risk of bias summary table; Description of data: risk of bias summary table. [file 12887_2020_2370_MOESM4_ESM.docx]
