# Supplementary material for: A sex/gender perspective on interventions to promote children’s and adolescents’ overall physical activity: results from genEffects systematic review
Source: BMC Pediatr. 2020 Oct 10;20:473. doi: 10.1186/s12887-020-02370-9 (PMC7547493; doi:10.1186/s12887-020-02370-9)
Supplement: Supplementary file 5 — Additional file 5. Intervention effects in relation to considerations of sex/gender in the included studies; Description of data: relation of intervention effects for each outcome for overall PA with regard to sex/gender by considering the results of the sex/gender checklist which indicated the extent with which studies have taken sex/gender into account. [file 12887_2020_2370_MOESM5_ESM.docx]

# Additional file 5: Intervention effects in relation to considerations of sex/gender in the included studies

1. **Studies with the same/similar significant intervention effects in both, boys and girls (35 Outcomes)**

|  |  | **Items of sex/gender checklist** | | | | | | | | | | **Sum of ratings** | | | | | | |
| --- | --- | --- | --- | --- | --- | --- | --- | --- | --- | --- | --- | --- | --- | --- | --- | --- | --- | --- |
| **Author, year of publication** | **Outcome** | **1** | **2** | **3** | **4** | **5** | **6** | **7** | **8** | **9** | **10** | **detailed** | | **basic** | | **no info** | | **poor** |
| Aburto et al., 2011 | steps/day |  |  |  |  |  |  |  |  |  |  | 1 | | 1 | | 7 | | 1 |
| Aceves-Martins et al., 2017 | total PA |  |  |  |  |  |  |  |  |  |  | 2 | | 2 | | 6 | | 0 |
| Annesi et al., 2015 | MVPA |  |  |  |  |  |  |  |  |  |  | 2 | | 3 | | 5 | | 0 |
|  | VPA |  |  |  |  |  |  |  |  |  |  |  |  |  |  |  |  |  |
| Beets et al., 2015 | MVPA |  |  |  |  |  |  |  |  |  |  | 3 | | 1 | | 5 | | 1 |
| Bryant et a., 2016 | steps/day |  |  |  |  |  |  |  |  |  |  | 2 | | 1 | | 7 | | 0 |
| Cronholm et al., 2017 | total PA |  |  |  |  |  |  |  |  |  |  | 2 | | 3 | | 5 | | 0 |
| Cruz et al., 2016 | engagement in PA |  |  |  |  |  |  |  |  |  |  | 1 | | 3 | | 6 | | 0 |
| Duncan et al., 2011 | steps/day |  |  |  |  |  |  |  |  |  |  | 1 | | 3 | | 6 | | 0 |
| Filho et al., 2016 | MVPA |  |  |  |  |  |  |  |  |  |  | 1 | | 3 | | 6 | | 0 |
| Greening et al., 2011 | total PA |  |  |  |  |  |  |  |  |  |  | 1 | | 2 | | 6 | | 1 |
| Haerens et al., 2007 (a) | LPA |  |  |  |  |  |  |  |  |  |  | 3 | | 2 | | 5 | | 0 |
|  | MVPA |  |  |  |  |  |  |  |  |  |  |  |  |  |  |  |  |  |
| Jago et al., 2014 | total PA |  |  |  |  |  |  |  |  |  |  | 2 | | 0 | | 7 | | 1 |
| Mehtälä et al., 2018 | LPA |  |  |  |  |  |  |  |  |  |  | 2 | | 1 | | 6 | | 1 |
| Jemmott et al., 2011 | meeting PA guidelines |  |  |  |  |  |  |  |  |  |  | 0 | | 4 | | 5 | | 1 |
| López-Fernández et al., 2016 | steps/min |  |  |  |  |  |  |  |  |  |  | 1 | | 2 | | 6 | | 1 |
| Morgan et al., 2011 | steps/day |  |  |  |  |  |  |  |  |  |  | 1 | | 2 | | 7 | | 0 |
| Morris et al., 2013 | steps/day |  |  |  |  |  |  |  |  |  |  | 3 | | 1 | | 5 | | 1 |
| Murrillo Pardo et al., 2016 | MVPA |  |  |  |  |  |  |  |  |  |  | 2 | | 1 | | 7 | | 0 |
| O’Dwyer et al., 2012 | total PA |  |  |  |  |  |  |  |  |  |  | 1 | | 2 | | 6 | | 1 |
| Pardo et al., 2014 | MVPA |  |  |  |  |  |  |  |  |  |  | 4 | | 2 | | 4 | | 0 |
| Peñalvo et al.; 2013 | PA habits (score) |  |  |  |  |  |  |  |  |  |  | 1 | | 0 | | 8 | | 1 |
| Razak et al., 2018 | MVPA |  |  |  |  |  |  |  |  |  |  | 1 | | 4 | | 5 | | 0 |
| Salminen et al., 2005 | frequency of PA (for girls)/exertion of PA (for boys) |  | 3 | 3 |  |  | 3 | 3 |  |  | 3 | 1 | | 2 | | 7 | | 0 |
| Sigmund et al., 2012 | steps/day |  |  |  |  |  |  |  |  |  |  | 6 | | 2 | | 1 | | 1 |
| Smpokos et al., 2010 | MVPA |  |  |  |  |  |  |  |  |  |  | 2 | | 1 | | 6 | | 1 |
| Stevens et al., 2003 | total PA |  |  |  |  |  |  |  |  |  |  | 1 | | 3 | | 6 | | 0 |
| Sutherland et al., 2016 (a) | MVPA |  |  |  |  |  |  |  |  |  |  | 1 | | 2 | | 6 | | 1 |
| Sutherland et al., 2016 (b) | MVPA |  |  |  |  |  |  |  |  |  |  | 3 | | 0 | | 6 | | 1 |
| Verstraete et al., 2007 | MPA |  |  |  |  |  |  |  |  |  |  | 1 | | 6 | | 3 | | 0 |
|  | MVPA |  |  |  |  |  |  |  |  |  |  |  |  |  |  |  |  |  |
| Weaver et al., 2017 | meeting PA guidelines |  |  |  |  |  |  |  |  |  |  | 2 | | 1 | | 6 | | 1 |
|  | MVPA |  |  |  |  |  |  |  |  |  |  |  |  |  |  |  |  |  |
| **% of ratings for each item** | |  |  |  |  |  |  |  |  |  |  |  | | | | | | |
| - detailed | | 0.0 | 0.0 | 3.3 | 3.3 | 0.0 | 3.3 | 3.3 | 36.7 | 93.3 | 33.3 |  |  |  |  |  |  |  |
| - basic | | 50.0 | 26.7 | 0.0 | 3.3 | 3.3 | 6.7 | 6.7 | 56.7 | 6.7 | 40.0 |  |  |  |  |  |  |  |
| - no information | | 0.0 | 73.3 | 96.7 | 93.3 | 96.7 | 90.0 | 90.0 | 6.7 | 0.0 | 26.7 |  |  |  |  |  |  |  |
| - poor (only for item 1) | | 50.0 |  |  |  |  |  |  |  |  |  |  |  |  |  |  |  |  |
| **Average number of ratings** | | | | | | | | | | | | 2.8 | 2.0 | | 5.7 | | 0.5 | |

*Note:* LPA=light physical activity; MPA=moderate physical activity; MVPA=moderate to vigorous physical activity; PA=physical activity; VPA=vigorous physical activity; ◼= detailed; ◼= basic; ◼= no information provided; ◼= poor.

1. **Studies with no significant intervention effects in both, boys and girls (70 outcomes)**

|  |  |  | **Items of sex/gender checklist** | | | | | | | | | | **Sum of ratings** | | | |
| --- | --- | --- | --- | --- | --- | --- | --- | --- | --- | --- | --- | --- | --- | --- | --- | --- |
| **Author, year of publication** | **Outcome** | | **1** | **2** | **3** | **4** | **5** | **6** | **7** | **8** | **9** | **10** | **detailed** | **basic** | **no info** | **poor** |
| Adab et al., 2018 (a) | MVPA | |  |  |  |  |  |  |  |  |  |  | 1 | 3 | 6 | 0 |
| Adab et al., 2018 (b) | MVPA | |  |  |  |  |  |  |  |  |  |  | 1 | 3 | 6 | 0 |
| Andrade et al., 2014 | total PA | |  |  |  |  |  |  |  |  |  |  | 1 | 3 | 6 | 0 |
|  | LPA | |  |  |  |  |  |  |  |  |  |  |  |  |  |  |
|  | MVPA | |  |  |  |  |  |  |  |  |  |  |  |  |  |  |
| Annesi et al., 2015 | MPA | |  |  |  |  |  |  |  |  |  |  | 2 | 3 | 5 | 0 |
|  | LPA | |  |  |  |  |  |  |  |  |  |  |  |  |  |  |
| Bhave et al., 2016 | total PA | |  |  |  |  |  |  |  |  |  |  | 2 | 3 | 5 | 0 |
| Black et al., 2010 | total PA | |  |  |  |  |  |  |  |  |  |  | 1 | 3 | 6 | 0 |
| Caballero et al., 2003 | total PA | |  |  |  |  |  |  |  |  |  |  | 2 | 1 | 7 | 0 |
| Carson et al., 2014 | MVPA | |  |  |  |  |  |  |  |  |  |  | 0 | 2 | 7 | 1 |
| Cui et al., 2012 | MVPA | |  |  |  |  |  |  |  |  |  |  | 0 | 4 | 5 | 1 |
| De Craemer et al., 2014 | MVPA | |  |  |  |  |  |  |  |  |  |  | 2 | 4 | 4 | 0 |
|  | VPA | |  |  |  |  |  |  |  |  |  |  |  |  |  |  |
|  | total PA | |  |  |  |  |  |  |  |  |  |  |  |  |  |  |
|  | MPA | |  |  |  |  |  |  |  |  |  |  |  |  |  |  |
| Fairclough et al., 2013 | MVPA | |  |  |  |  |  |  |  |  |  |  | 1 | 1 | 7 | 1 |
|  | VPA | |  |  |  |  |  |  |  |  |  |  |  |  |  |  |
| Gomez et al., 2018 | total PA | |  |  |  |  |  |  |  |  |  |  | 1 | 2 | 7 | 0 |
| Goran et al., 2005 | LPA | |  |  |  |  |  |  |  |  |  |  | 2 | 2 | 6 | 0 |
| Gorely et al., 2011 | MVPA | |  |  |  |  |  |  |  |  |  |  | 1 | 1 | 7 | 1 |
|  | steps/day | |  |  |  |  |  |  |  |  |  |  |  |  |  |  |
| Grasten et al., 2018 | MVPA | |  |  |  |  |  |  |  |  |  |  | 1 | 3 | 6 | 0 |
| Grydeland et al., 2013 | LPA | |  |  |  |  |  |  |  |  |  |  | 2 | 3 | 5 | 0 |
|  | MVPA | |  |  |  |  |  |  |  |  |  |  |  |  |  |  |
| Haerens et al., 2007 (a) | total PA | |  |  |  |  |  |  |  |  |  |  | 3 | 2 | 5 | 0 |
|  | meeting PA guidelines | |  |  |  |  |  |  |  |  |  |  |  |  |  |  |
| Haerens et al., 2007 (b) | total PA | |  |  |  |  |  |  |  |  |  |  | 1 | 3 | 6 | 0 |
| Hovell et al., 2009 | VPA | |  |  |  |  |  |  |  |  |  |  | 1 | 3 | 6 | 0 |
| Jago et al., 2011 | MVPA | |  |  |  |  |  |  |  |  |  |  | 1 | 2 | 6 | 1 |
| Jago et al., 2014 | MVPA | |  |  |  |  |  |  |  |  |  |  | 2 | 0 | 7 | 1 |
| Kobel et al., 2014 | MVPA | |  |  |  |  |  |  |  |  |  |  | 1 | 3 | 6 | 0 |
| Lanckriet et al., 2017 | total PA | |  |  |  |  |  |  |  |  |  |  | 0 | 3 | 7 | 0 |
|  | MPA | |  |  |  |  |  |  |  |  |  |  |  |  |  |  |
|  | LPA | |  |  |  |  |  |  |  |  |  |  |  |  |  |  |
|  | VPA | |  |  |  |  |  |  |  |  |  |  |  |  |  |  |
| Laukkanen et al., 2015 | MVPA | |  |  |  |  |  |  |  |  |  |  | 1 | 3 | 6 | 0 |
| Lawlor et al., 2016 | MVPA | |  |  |  |  |  |  |  |  |  |  | 2 | 3 | 5 | 0 |
| McNeil et al., 2009 | overall PA | |  |  |  |  |  |  |  |  |  |  | 1 | 2 | 6 | 1 |
| Mehtälä et al., 2018 | total PA | |  |  |  |  |  |  |  |  |  |  | 2 | 1 | 6 | 1 |
| Meier et al., 2007 | steps/day | |  |  |  |  |  |  |  |  |  |  | 1 | 3 | 6 | 0 |
| Meyer et al., 2014 | total PA | |  |  |  |  |  |  |  |  |  |  | 1 | 1 | 7 | 1 |
|  | MVPA | |  |  |  |  |  |  |  |  |  |  |  |  |  |  |
| Ni Mhurchu et al., 2008 | total PA | |  |  |  |  |  |  |  |  |  |  | 0 | 2 | 7 | 1 |
|  | MPA | |  |  |  |  |  |  |  |  |  |  |  |  |  |  |
|  | VPA | |  |  |  |  |  |  |  |  |  |  |  |  |  |  |
|  | PAQ-Score | |  |  |  |  |  |  |  |  |  |  |  |  |  |  |
| Nyberg et al., 2015 | total PA | |  |  |  |  |  |  |  |  |  |  | 2 | 2 | 6 | 1 |
|  | MVPA | |  |  |  |  |  |  |  |  |  |  |  |  |  |  |
| Nyberg et al., 2016 | total PA | |  |  |  |  |  |  |  |  |  |  | 1 | 2 | 7 | 0 |
|  | MVPA | |  |  |  |  |  |  |  |  |  |  |  |  |  |  |
| Patrick et al., 2006 | MVPA | |  |  |  |  |  |  |  |  |  |  | 2 | 3 | 5 | 0 |
| Reilly et al., 2006 | total PA | |  |  |  |  |  |  |  |  |  |  | 3 | 2 | 6 | 0 |
| Salmon et al., 2010 | MVPA | |  |  |  |  |  |  |  |  |  |  | 1 | 3 | 6 | 0 |
| Tarp et al., 2016 | total PA | |  |  |  |  |  |  |  |  |  |  | 3 | 2 | 6 | 1 |
|  | MVPA | |  |  |  |  |  |  |  |  |  |  |  |  |  |  |
| Telford et al., 2016 | steps/day | |  |  |  |  |  |  |  |  |  |  | 3 | 1 | 5 | 1 |
|  | MVPA | |  |  |  |  |  |  |  |  |  |  |  |  |  |  |
| Toftager et al., 2014 | total PA | |  |  |  |  |  |  |  |  |  |  | 3 | 3 | 5 | 0 |
|  | MVPA | |  |  |  |  |  |  |  |  |  |  |  |  |  |  |
| Vašíčková et al.; 2013 | total PA | |  |  |  |  |  |  |  |  |  |  | 3 | 1 | 5 | 1 |
| Verbestel et al., 2013 | total PA | |  |  |  |  |  |  |  |  |  |  | 2 | 1 | 7 | 0 |
|  | LPA | |  |  |  |  |  |  |  |  |  |  |  |  |  |  |
|  | MVPA | |  |  |  |  |  |  |  |  |  |  |  |  |  |  |
| Verloigne et al., 2012 | LPA | |  |  |  |  |  |  |  |  |  |  | 2 | 3 | 5 | 0 |
|  | MVPA | |  |  |  |  |  |  |  |  |  |  |  |  |  |  |
| Verstraete et al., 2007 | LPA | |  |  |  |  |  |  |  |  |  |  | 2 | 5 | 3 | 0 |
|  | VPA | |  |  |  |  |  |  |  |  |  |  |  |  |  |  |
|  | LPA | |  |  |  |  |  |  |  |  |  |  |  |  |  |  |
| Wang et al., 2018 | MVPA | |  |  |  |  |  |  |  |  |  |  | 2 | 1 | 6 | 1 |
| **% of ratings for each item** | | |  |  |  |  |  |  |  |  |  |  |  |  |  |  |
| - detailed | | | 0.0 | 2.3 | 0.0 | 9.3 | 0.0 | 9.3 | 0.0 | 30.2 | 83.7 | 18.6 |  |  |  |  |
| - basic | | | 65.1 | 32.6 | 0.0 | 0.0 | 2.3 | 0.0 | 4.7 | 53.5 | 16.3 | 44.2 |  |  |  |  |
| - no information | | | 0.0 | 65.1 | 100.0 | 90.7 | 97.7 | 97.7 | 95.3 | 16.3 | 0.0 | 37.2 |  |  |  |  |
| - poor (only for item 1) | | | 34.9 |  |  |  |  |  |  |  |  |  |  |  |  |  |
| **Average number of ratings** | | | | | | | | | | | | | 1.5 | 2.3 | 5.9 | 0.3 |

*Note:* LPA=light physical activity; MPA=moderate physical activity; MVPA=moderate to vigorous physical activity; PA=physical activity; VPA=vigorous physical activity; PAQ=physical activity Questionnaire; ◼= detailed; ◼= basic; ◼= no information provided; ◼= poor.

1. **Studies with different intervention effects in boys and girls (15 outcomes)**

|  | |  |  | **Item of sex/gender checklist** | | | | | | | | | | **Sum of ratings** | | | | | |
| --- | --- | --- | --- | --- | --- | --- | --- | --- | --- | --- | --- | --- | --- | --- | --- | --- | --- | --- | --- |
| **Author, year of publication** | | **Outcome** | **Intervention effect in favour of** | **1** | **2** | **3** | **4** | **5** | **6** | **7** | **8** | **9** | **10** | **detailed** | | **basic** | **no info** | **poor** | |
| Goran et al., 2005 | | total PA | ♂ |  |  |  |  |  |  |  |  |  |  | 2 | | 2 | 6 | 0 | |
|  |  | MPA | ♂ |  |  |  |  |  |  |  |  |  |  |  |  |  |  |  |  |
|  |  | VPA | ♂ |  |  |  |  |  |  |  |  |  |  |  |  |  |  |  |  |
|  |  | MVPA | ♂ |  |  |  |  |  |  |  |  |  |  |  |  |  |  |  |  |
| Grydeland et al., 2013 | | total PA | ♀ |  |  |  |  |  |  |  |  |  |  | 1 | | 3 | 6 | 0 | |
| Gutherie et al., 2015 | | MVPA | ♂ |  |  |  |  |  |  |  |  |  |  | 1 | | 2 | 6 | 1 | |
| Manios et al., 2006 | | MVPA | ♂ |  |  |  |  |  |  |  |  |  |  | 1 | | 3 | 6 | 0 | |
| Mehtälä et al., 2018 | | MVPA | ♀ |  |  |  |  |  |  |  |  |  |  | 2 | | 1 | 6 | 1 | |
|  |  | LMVPA | ♀ |  |  |  |  |  |  |  |  |  |  |  |  |  |  |  |  |
|  |  | VPA | ♂ |  |  |  |  |  |  |  |  |  |  |  |  |  |  |  |  |
|  |  | meeting PA guidelines | ♂ |  |  |  |  |  |  |  |  |  |  |  |  |  |  |  |  |
| Morris et al., 2013 | | MVPA | ♂ |  |  |  |  |  |  |  |  |  |  | 2 | | 2 | 5 | 1 | |
| Pienaar et al., 2012 | | total PA | ♂ |  |  |  |  |  |  |  |  |  |  | 1 | | 5 | 4 | 0 | |
| Salmon et al., 2008 | | total PA | ♂ |  |  |  |  |  |  |  |  |  |  | 1 | | 2 | 6 | 1 | |
| Sanaeinasab et al., 2012 | | total PA | ♂ |  |  |  |  |  |  |  |  |  |  | 1 | | 2 | 6 | 1 | |
| **% of ratings for each item** | | |  |  |  |  |  |  |  |  |  |  |  | | | | | |  |
| - detailed | | | 0.0 | 0.0 | 0.0 | 0.0 | 0.0 | 0.0 | 0.0 | 22.2 | 100.0 | 11.1 |  |  |  |  |  |  |  |
| - basic | | | 44.4 | 22.2 | 0.0 | 0.0 | 0.0 | 0.0 | 11.1 | 77.8 | 0.0 | 88.9 |  |  |  |  |  |  |  |
| - no information | | | 0.0 | 77.8 | 100.0 | 100.0 | 100.0 | 100.0 | 88.9 | 0.0 | 0.0 | 0.0 |  |  |  |  |  |  |  |
| - poor (only for item 1) | | | 55.6 |  |  |  |  |  |  |  |  |  |  |  |  |  |  |  |  |
| **Average number of ratings** | | | | | | | | | | | | | | 1.3 | 2.4 | | 5.7 | | 0.6 |

*Note:* LMVPA=light to moderate to vigorous physical activity; LPA=light physical activity; MPA=moderate physical activity; MVPA=moderate to vigorous physical activity; PA=physical activity; VPA=vigorous physical activity; ◼= detailed; ◼= basic; ◼= no information provided; ◼= poor.

1. **Single sex/gender studies**
   1. **Single sex/gender studies with sig. intervention effects (in favour of the intervention group) (10 Outcomes)**

|  |  |  | **Item of sex/gender checklist** | | | | | | | | | | **Sum of ratings** | | | | |
| --- | --- | --- | --- | --- | --- | --- | --- | --- | --- | --- | --- | --- | --- | --- | --- | --- | --- |
| **Author, year of publication** | **Outcome** | **Sex/gender** | **1** | **2** | **3** | **4** | **5** | **6** | **7** | **8** | **9** | **10** | **detailed** | **basic** | **no info** | **poor** | **not relevant** |
| Darabi et al., 2017 | total PA | ♀ |  | 1 | 3 | 4 | 4 | 3 | 3 | 4 | 4 | 3 | 0 | 1 | 5 | 0 | 4 |
| Dunton et al., 2007 | VPA | ♀ |  | 1 | 3 | 4 | 4 | 3 | 3 | 4 | 4 | 1 | 0 | 2 | 4 | 0 | 4 |
| Ghaffari et al., 2013 | total PA | ♂ |  |  |  |  |  |  |  |  |  |  | 0 | 1 | 5 | 0 | 4 |
| Morgan et al., 2018 | steps/day | ♀ |  | 1 | 3 | 4 | 4 | 2 | 1 | 4 | 4 | 1 | 1 | 4 | 1 | 0 | 4 |
| Pate et al., 2005 | VPA | ♀ |  | 1 | 3 |  | 4 | 1 | 3 | 4 | 4 | 1 | 0 | 4 | 2 | 0 | 4 |
| Rostami-Moez et al., 2017 | total PA | ♀ |  | 1 | 3 | 4 | 4 | 3 | 3 | 4 | 4 | 2 | 1 | 2 | 3 | 0 | 4 |
| Schneider et al., 2008 | VPA | ♀ |  |  | 3 | 4 | 4 |  | 3 | 4 | 4 | 1 | 0 | 2 | 4 | 0 | 4 |
| Schneider et al., 2011 | MVPA | ♀ |  | 1 | 3 | 4 | 4 | 3 | 3 | 4 | 4 | 1 | 0 | 2 | 4 | 0 | 4 |
| Schofield et al., 2005 | steps/day | ♀ |  | 1 | 3 | 4 | 4 | 3 | 1 | 4 | 4 | 2 | 1 | 2 | 3 | 0 | 4 |
| **% of ratings for each item** | | |  |  |  |  |  |  |  |  |  |  |  |  |  |  |  |
| - detailed | | | 0.0 | 0.0 | 0.0 | 0.0 | 0.0 | 11.1 | 0.0 | 0.0 | 0.0 | 22.2 |  |  |  |  |  |
| - basic | | | 44.4 | 88.9 | 0.0 | 0.0 | 0.0 | 11.1 | 22.2 | 0.0 | 0.0 | 55.6 |  |  |  |  |  |
| - no information | | | 55.6 | 11.1 | 100.0 | 0.0 | 0.0 | 77.8 | 77.8 | 0.0 | 0.0 | 22.2 |  |  |  |  |  |
| - poor (only for item 1) | | | 0.0 |  |  |  |  |  |  |  |  |  |  |  |  |  |  |
| **Average number of ratings** | | | | | | | | | | | | | 0.4 | 2.0 | 3.5 | 0.1 | 4.0 |

*Note*: ♀=only girls; ♂=only boys; LPA=light physical activity; MPA=moderate physical activity; MVPA=moderate to vigorous physical activity; PA=physical activity; VPA=vigorous physical activity; ◼= detailed; ◼= basic; ◼= no information provided; ◼= poor; ◼ = not relevant.

- 1. **Single sex/gender studies without sig. intervention effects (34 Outcomes)**

|  |  |  | **Item of sex/gender checklist** | | | | | | | | | | **Sum of ratings** | | | | |
| --- | --- | --- | --- | --- | --- | --- | --- | --- | --- | --- | --- | --- | --- | --- | --- | --- | --- |
| **Author, year of publication** | **Outcome** | **Sex/gender** | **1** | **2** | **3** | **4** | **5** | **6** | **7** | **8** | **9** | **10** | **detailed** | **basic** | **no info** | **poor** | **not relevant** |
| Bakhoya et al.; 2016 | LPA | ♀ |  | 3 | 3 | 4 | 4 | 3 | 3 | 4 | 4 | 3 | 0 | 0 | 6 | 0 | 4 |
|  | MVPA |  |  |  |  |  |  |  |  |  |  |  |  |  |  |  |  |
| Dewar et al., 2014 | MPA | ♀ |  | 1 | 3 | 4 | 4 | 3 | 3 | 4 | 4 | 2 | 1 | 1 | 4 | 0 | 4 |
|  | VPA | ♀ |  |  |  |  |  |  |  |  |  |  |  |  |  |  |  |
|  | MVPA |  |  |  |  |  |  |  |  |  |  |  |  |  |  |  |  |
| Dubuy et al., 2014 | total PA | ♂ |  |  |  |  |  |  |  |  |  |  | 1 | 3 | 2 | 0 | 4 |
| Hardman et al., 2009 | steps/day | ♀ |  | 1 | 3 | 4 | 4 | 3 | 3 | 4 | 4 | 1 | 0 | 2 | 4 | 0 | 4 |
| Klesges et al., 2010 | total PA | ♀ |  | 1 | 3 | 4 | 4 | 3 | 3 | 4 | 4 | 1 | 0 | 3 | 3 | 0 | 4 |
|  | MVPA |  |  |  |  |  |  |  |  |  |  |  |  |  |  |  |  |
| Lubans et al., 2011 | steps/day | ♂ |  |  |  |  |  |  |  |  |  |  | 0 | 2 | 4 | 0 | 4 |
| Lubans et al., 2012 | total PA | ♀ |  | 1 | 3 | 4 | 4 | 3 | 3 | 4 | 4 | 2 | 1 | 2 | 3 | 0 | 4 |
|  | MVPA |  |  |  |  |  |  |  |  |  |  |  |  |  |  |  |  |
| Lubans et al., 2016 | total PA | ♂ |  |  |  |  |  |  |  |  |  |  | 0 | 2 | 3 | 1 | 4 |
|  | MVPA |  |  |  |  |  |  |  |  |  |  |  |  |  |  |  |  |
| Okely et al., 2017 | total PA | ♀ |  | 1 | 3 | 4 | 4 | 1 | 3 | 4 | 4 | 2 | 1 | 2 | 3 | 0 | 4 |
|  | LPA |  |  |  |  |  |  |  |  |  |  |  |  |  |  |  |  |
|  | MPA |  |  |  |  |  |  |  |  |  |  |  |  |  |  |  |  |
|  | VPA |  |  |  |  |  |  |  |  |  |  |  |  |  |  |  |  |
|  | MVPA |  |  |  |  |  |  |  |  |  |  |  |  |  |  |  |  |
| Pate et al., 2005 | MVPA | ♀ |  | 1 | 3 |  | 4 | 1 | 3 | 4 | 4 | 1 | 0 | 4 | 2 | 0 | 4 |
| Robbins et al., 2012 | MVPA | ♀ |  | 1 | 3 | 4 | 4 | 3 | 3 | 4 | 4 | 2 | 1 | 1 | 4 | 0 | 4 |
| Robbins et al., 2018 | MVPA | ♀ |  | 1 | 3 | 4 | 4 | 3 | 3 | 4 | 4 | 2 | 1 | 1 | 4 | 0 | 4 |
| Rosenkranz et al., 2014 | MVPA | ♀ |  | 3 | 1 | 4 | 4 | 3 | 3 | 4 | 4 | 2 | 1 | 1 | 3 | 1 | 4 |
| Schneider et al., 2011 | VPA | ♀ |  | 1 | 3 | 4 | 4 | 3 | 3 | 4 | 4 | 1 | 0 | 2 | 4 | 0 | 4 |
| Schofield et al., 2005 | MVPA | ♀ |  |  |  |  |  |  |  |  |  |  | 1 | 2 | 3 | 0 | 4 |
|  | VPA |  |  |  |  |  |  |  |  |  |  |  |  |  |  |  |  |
| Sebire et al., 2018 | MVPA | ♀ |  | 1 | 3 | 4 | 4 | 3 | 3 | 4 | 4 | 2 | 1 | 1 | 3 | 1 | 4 |
| Smith et al., 2014 | total PA | ♂ |  |  |  |  |  |  |  |  |  |  | 1 | 3 | 2 | 0 | 4 |
|  | MVPA |  |  |  |  |  |  |  |  |  |  |  |  |  |  |  |  |
| Story et al., 2003 | total PA | ♀ |  | 1 | 3 |  | 4 | 2 | 3 | 4 | 4 | 1 | 1 | 2 | 2 | 1 | 4 |
|  | MVPA |  |  |  |  |  |  |  |  |  |  |  |  |  |  |  |  |
| Taymoori et al., 2008 | MVPA | ♀ |  | 2 | 1 | 4 | 4 | 3  3 | 1 | 4 | 4 | 2 | 2 | 2 | 2 | 0 | 4 |
|  | total PA |  |  |  |  |  |  |  |  |  |  |  |  |  |  |  |  |
| **% of ratings for each item** | | |  |  |  |  |  |  |  |  |  |  |  |  |  |  |  |
| - detailed | | | 0.0 | 5.3 | 10.5 | 0.0 | 0.0 | 15.8 | 0.0 | 0.0 | 0.0 | 47.4 |  |  |  |  |  |
| - basic | | | 31.6 | 84.2 | 0.0 | 0.0 | 0.0 | 10.5 | 10.5 | 0.0 | 0.0 | 42.1 |  |  |  |  |  |
| - no information | | | 47.4 | 10.5 | 89.5 | 0.0 | 0.0 | 73.7 | 89.5 | 0.0 | 0.0 | 10.5 |  |  |  |  |  |
| - poor (only for item 1) | | | 21.1 |  |  |  |  |  |  |  |  |  |  |  |  |  |  |
| **Average number of ratings** | | | | | | | | | | | | | 0.7 | 1.8 | 3.3 | 0.3 | 4.0 |

*Note*: ♀=only girls; ♂=only boys; MVPA=moderate to vigorous physical activity; PA=physical activity; VPA=vigorous physical activity; ◼= detailed; ◼= basic; ◼= no information provided; ◼= poor; ◼= not relevant.

**Summary of all tables:**

|  | **% of ratings of items of sex/gender checklist** | | | | | | | | | |
| --- | --- | --- | --- | --- | --- | --- | --- | --- | --- | --- |
|  | **1** | **2** | **3** | **4** | **5** | **6** | **7** | **8** | **9** | **10** |
| **Detailed** |  |  |  |  |  |  |  |  |  |  |
| - Studies with the same/similar significant intervention effects in both, boys and girls | 0.0 | 0.0 | 3.3 | 3.3 | 0.0 | 3.3 | 3.3 | 36.7 | 93.3 | 33.3 |
| - Studies with no significant intervention effects in both, boys and girls | 0.0 | 2.3 | 0.0 | 9.3 | 0.3 | 9.3 | 0.0 | 30.2 | 83.7 | 18.6 |
| - Studies with different intervention effects in boys and girls | 0.0 | 0.0 | 0.0 | 0.0 | 0.0 | 0.0 | 0.0 | 22.2 | 100.0 | 11.1 |
| - Single sex/gender studies with sig. intervention effects (in favour of the intervention group) | 0.0 | 0.0 | 0.0 | * | * | 11.1 | 0.0 | * | * | 22.2 |
| - Single sex/gender studies without sig. intervention effects | 0.0 | 5.3 | 10.5 | * | * | 15.8 | 0.0 | * | * | 47.4 |
| **Basic** |  |  |  |  |  |  |  |  |  |  |
| - Studies with the same/similar significant intervention effects in both, boys and girls | 50.0 | 26.7 | 0.0 | 3.3 | 3.3 | 6.7 | 6.7 | 56.7 | 6.7 | 40.0 |
| - Studies with no significant intervention effects in both, boys and girls | 65.1 | 32.6 | 0.0 | 0.0 | 2.3 | 0.0 | 4.7 | 53.5 | 16.3 | 44.2 |
| - Studies with different intervention effects in boys and girls | 44.4 | 22.2 | 0.0 | 0.0 | 0.0 | 0.0 | 11.1 | 77.8 | 0.0 | 88.9 |
| - Single sex/gender studies with sig. intervention effects (in favour of the intervention group) | 44.4 | 88.9 | 0.0 | * | * | 11.1 | 22.2 | * | * | 55.6 |
| - Single sex/gender studies without sig. intervention effects | 31.6 | 84.2 | 0.0 | * | * | 10.5 | 10.5 | * | * | 42.1 |
| **No information provided** |  |  |  |  |  |  |  |  |  |  |
| - Studies with the same/similar significant intervention effects in both, boys and girls | 0.0 | 73.3 | 96.7 | 93.3 | 96.7 | 90.0 | 90.0 | 6.7 | 0.0 | 26.7 |
| - Studies with no significant intervention effects in both, boys and girls | 0.0 | 65.1 | 100.0 | 90.7 | 97.7 | 90.7 | 95.3 | 16.3 | 0.0 | 37.2 |
| - Studies with different intervention effects in boys and girls | 0.0 | 77.8 | 100.0 | 100.0 | 100.0 | 100.0 | 88.9 | 0.0 | 0.0 | 0.0 |
| - Single sex/gender studies with sig. intervention effects (in favour of the intervention group) | 55.6 | 11.1 | 100.0 | * | * | 77.8 | 77.8 | * | * | 22.2 |
| - Single sex/gender studies without sig. intervention effects | 47.4 | 10.5 | 89.5 | * | * | 73.7 | 89.5 | * | * | 10.5 |
| **Poor **** |  |  |  |  |  |  |  |  |  |  |
| - Studies with the same/similar significant intervention effects in both, boys and girls | 50.0 |  |  |  |  |  |  |  |  |  |
| - Studies with no significant intervention effects in both, boys and girls | 34.9 |  |  |  |  |  |  |  |  |  |
| - Studies with different intervention effects in boys and girls | 55.6 |  |  |  |  |  |  |  |  |  |
| - Single sex/gender studies with sig. intervention effects (in favour of the intervention group) | 0.0 |  |  |  |  |  |  |  |  |  |
| - Single sex/gender studies without sig. intervention effects | 21.1 |  |  |  |  |  |  |  |  |  |

*Note:* *not relevant (for single sex/gender studies); ** only for item 1

**References**

Aburto, N. J., Fulton, J. E., Safdie, M., Duque, T., Bonvecchio, A., & Rivera, J. A. (2011). Effect of a school-based intervention on physical activity: cluster-randomized trial. *Medicine and Science in Sports and Exercise, 43*(10), 1898-1906. doi:10.1249/MSS.0b013e318217ebec

Aceves-Martins, M., Llaurado, E., Tarro, L., Morina, D., Papell-Garcia, I., Prades-Tena, J., Kettner-Hoeberg, H., Puiggros, F., Arola, L., Davies, A., Giralt, M., & Sola, R. (2017). A School-Based, Peer-Led, Social Marketing Intervention To Engage Spanish Adolescents in a Healthy Lifestyle ("We Are Cool"-Som la Pera Study): A Parallel-Cluster Randomized Controlled Study. *Childhood Obesity, 13*(4), 300-313. doi:10.1089/chi.2016.0216

Adab, P., Barrett, T., Bhopal, R., Cade, J. E., Canaway, A., Cheng, K. K., Clarke, J., Daley, A., Deeks, J., Duda, J., Ekelund, U., Frew, E., Gill, P., Griffin, T., Hemming, K., Hurley, K., Lancashire, E. R., Martin, J., McGee, E., Pallan, M. J., Parry, J., & Passmore, S. (2018a). The West Midlands ActiVe lifestyle and healthy Eating in School children (WAVES) study: a cluster randomised controlled trial testing the clinical effectiveness and cost-effectiveness of a multifaceted obesity prevention intervention programme targeted at children aged 6-7 years. *Health Technology Assessment, 22*(8), 1-608. doi:10.3310/hta22080

Adab, P., Pallan, M. J., Lancashire, E. R., Hemming, K., Frew, E., Barrett, T., Bhopal, R., Cade, J. E., Canaway, A., Clarke, J. L., Daley, A., Deeks, J. J., Duda, J. L., Ekelund, U., Gill, P., Griffin, T., McGee, E., Hurley, K., Martin, J., Parry, J., Passmore, S., & Cheng, K. K. (2018b). Effectiveness of a childhood obesity prevention programme delivered through schools, targeting 6 and 7 year olds: cluster randomised controlled trial (WAVES study).[Erratum appears in BMJ. 2018 May 2;361:k1954; PMID: 29720463]. *BMJ, 360*, k211. doi:10.1136/bmj.k211

Andrade, S., Lachat, C., Ochoa-Aviles, A., Verstraeten, R., Huybregts, L., Roberfroid, D., Andrade, D., Camp, J. V., Rojas, R., Donoso, S., Cardon, G., & Kolsteren, P. (2014). A school-based intervention improves physical fitness in Ecuadorian adolescents: a cluster-randomized controlled trial. *International Journal of Behavioral Nutrition and Physical Activity, 11*, 153. doi:10.1186/s12966-014-0153-5

Annesi, J. J., & Vaughn, L. L. (2015). Evidence-Based Referral: Effects of the Revised "Youth Fit 4 Life" Protocol on Physical Activity Outputs. *Permanente Journal, 19*(3), 48-53. doi:10.7812/TPP/14-228

Bakhoya, M., Ling, J., Pfeiffer, K. A., & Robbins, L. B. (2016). Evaluating Mailed Motivational, Individually Tailored Postcard Boosters for Promoting Girls' Postintervention Moderate-to-Vigorous Physical Activity. *Nursing Research, 65*(5), 415-420. doi:10.1097/NNR.0000000000000173

Beets, M. W., Weaver, R. G., Turner-McGrievy, G., Huberty, J., Ward, D. S., Pate, R. R., Freedman, D., Hutto, B., Moore, J. B., & Beighle, A. (2015). Making policy practice in afterschool programs: a randomized controlled trial on physical activity changes. *American Journal of Preventive Medicine, 48*(6), 694-706. doi:10.1016/j.amepre.2015.01.012

Bhave, S., Pandit, A., Yeravdekar, R., Madkaikar, V., Chinchwade, T., Shaikh, N., Shaikh, T., Naik, S., Marley-Zagar, E., & Fall, C. H. (2016). Effectiveness of a 5-year school-based intervention programme to reduce adiposity and improve fitness and lifestyle in Indian children; the SYM-KEM study. *Archives of Disease in Childhood, 101*(1), 33-41. doi:10.1136/archdischild-2015-308673

Black, M. M., Hager, E. R., Le, K., Anliker, J., Arteaga, S. S., Diclemente, C., Gittelsohn, J., Magder, L., Papas, M., Snitker, S., Treuth, M. S., & Wang, Y. (2010). Challenge! Health promotion/obesity prevention mentorship model among urban, black adolescents. *Pediatrics, 126*(2), 280-288. doi:10.1542/peds.2009-1832

Bryant, E. S., Duncan, M. J., Birch, S. L., & James, R. S. (2016). Can Fundamental Movement Skill Mastery Be Increased via a Six Week Physical Activity Intervention to Have Positive Effects on Physical Activity and Physical Self-Perception? *Sports, 4*(1), 16. doi:10.3390/sports4010010

Caballero, B., Clay, T., Davis, S. M., Ethelbah, B., Rock, B. H., Lohman, T., Norman, J., Story, M., Stone, E. J., Stephenson, L., Stevens, J., & Pathways Study Research, G. (2003). Pathways: a school-based, randomized controlled trial for the prevention of obesity in American Indian schoolchildren. *American Journal of Clinical Nutrition, 78*(5), 1030-1038.

Carson, R. L., Castelli, D. M., Pulling Kuhn, A. C., Moore, J. B., Beets, M. W., Beighle, A., Aija, R., Calvert, H. G., & Glowacki, E. M. (2014). Impact of trained champions of comprehensive school physical activity programs on school physical activity offerings, youth physical activity and sedentary behaviors. *Preventive Medicine, 69*(S), S12-S19.

Cronholm, F., Rosengren, B. E., Karlsson, C., & Karlsson, M. K. (2017). A Physical Activity Intervention Program in School is Also Accompanied by Higher Leisure-Time Physical Activity: A Prospective Controlled 3-Year Study in 194 Prepubertal Children. *Journal of Physical Activity and Health, 14*(4), 301-307. doi:10.1123/jpah.2016-0213

Cruz, T. H., Davis, S. M., Myers, O. B., O'Donald, E. R., Sanders, S. G., & Sheche, J. N. (2016). Effects of an Obesity Prevention Intervention on Physical Activity Among Preschool Children: The CHILE Study. *Health Promotion Practice, 17*(5), 693-701. doi:10.1177/1524839916629974

Cui, Z., Shah, S., Yan, L., Pan, Y., Gao, A., Shi, X., Wu, Y., & Dibley, M. J. (2012). Effect of a school-based peer education intervention on physical activity and sedentary behaviour in Chinese adolescents: a pilot study. *BMJ Open, 2*(3). doi:10.1136/bmjopen-2011-000721

Darabi, F., Kaveh, M. H., Majlessi, F., Farahani, F. K. A., Yaseri, M., & Shojaeizadeh, D. (2017). Effect of theory-based intervention to promote physical activity among adolescent girls: a randomized control trial. *Electronic Physician [Electronic Resource], 9*(4), 4238-4247. doi:10.19082/4238

De Craemer, M., De Decker, E., Verloigne, M., De Bourdeaudhuij, I., Manios, Y., Cardon, G., & ToyBox-study, g. (2014). The effect of a kindergarten-based, family-involved intervention on objectively measured physical activity in Belgian preschool boys and girls of high and low SES: the ToyBox-study. *International Journal of Behavioral Nutrition and Physical Activity, 11*(1), 38. doi:10.1186/1479-5868-11-38

Dewar, D. L., Morgan, P. J., Plotnikoff, R. C., Okely, A. D., Batterham, M., & Lubans, D. R. (2014). Exploring changes in physical activity, sedentary behaviors and hypothesized mediators in the NEAT girls group randomized controlled trial. *Journal of Science and Medicine in Sport, 17*(1), 39-46. doi:10.1016/j.jsams.2013.02.003

Dubuy, V., De Cocker, K., De Bourdeaudhuij, I., Maes, L., Seghers, J., Lefevre, J., De Martelaer, K., Brooke, H., & Cardon, G. (2014). Evaluation of a real world intervention using professional football players to promote a healthy diet and physical activity in children and adolescents from a lower socio-economic background: a controlled pretest-posttest design. *BMC Public Health, 14*, 457. doi:10.1186/1471-2458-14-457

Duncan, S., McPhee, J. C., Schluter, P. J., Zinn, C., Smith, R., & Schofield, G. (2011). Efficacy of a compulsory homework programme for increasing physical activity and healthy eating in children: the healthy homework pilot study. *International Journal of Behavioral Nutrition and Physical Activity, 8*, 127. doi:10.1186/1479-5868-8-127

Dunton, G. F., Schneider, M., & Cooper, D. M. (2007). An investigation of psychosocial factors related to changes in physical activity and fitness among female adolescents. *Psychology & Health, 22*(8), 929-944.

Fairclough, S. J., Hackett, A. F., Davies, I. G., Gobbi, R., Mackintosh, K. A., Warburton, G. L., Stratton, G., van Sluijs, E. M., & Boddy, L. M. (2013). Promoting healthy weight in primary school children through physical activity and nutrition education: a pragmatic evaluation of the CHANGE! randomised intervention study. *BMC Public Health, 13*, 626. doi:10.1186/1471-2458-13-626

Filho, V. C., da Silva, K. S., Mota, J., Beck, C., & da Silva Lopes, A. (2016). A Physical Activity Intervention for Brazilian Students From Low Human Development Index Areas: A Cluster-Randomized Controlled Trial. *Journal of Physical Activity and Health, 13*(11), 1174-1182. doi:10.1123/jpah.2016-0113

Ghaffari, M., Sharifirad, G., Malekmakan, E., & Hassanzadeh, A. (2013). Effect of educational intervention on physical activity-related knowledge, attitude and behavior of among first-grade students of male high schools. *Journal of Education & Health Promotion, 2*, 4. doi:10.4103/2277-9531.106642

Gomez, S. F., Casas Esteve, R., Subirana, I., Serra-Majem, L., Fletas Torrent, M., Homs, C., Bawaked, R. A., Estrada, L., Fito, M., & Schroder, H. (2018). Effect of a community-based childhood obesity intervention program on changes in anthropometric variables, incidence of obesity, and lifestyle choices in Spanish children aged 8 to 10 years. *European Journal of Pediatrics*, 19. doi:10.1007/s00431-018-3207-x

Goran, M. I., & Reynolds, K. (2005). Interactive Multimedia for Promoting Physical Activity (IMPACT) in Children. *Obesity Research, 13*(4), 762-771. doi:10.1038/oby.2005.86

Gorely, T., Morris, J. G., Musson, H., Brown, S., Nevill, A., & Nevill, M. E. (2011). Physical activity and body composition outcomes of the GreatFun2Run intervention at 20 month follow-up. *International Journal of Behavioral Nutrition and Physical Activity, 8*, 74. doi:10.1186/1479-5868-8-74

Gråstén, A., Watt, A., Liukkonen, J., & Jaakkola, T. (2017). Effects of School-Based Physical Activity Program on Students' Moderate-to-Vigorous Physical Activity and Perceptions of Physical Competence. *Journal of Physical Activity and Health, 14*(6), 455-464.

Greening, L., Harrell, K. T., Low, A. K., & Fielder, C. E. (2011). Efficacy of a school-based childhood obesity intervention program in a rural southern community: TEAM Mississippi Project. *Obesity, 19*(6), 1213-1219. doi:10.1038/oby.2010.329

Grydeland, M., Bergh, I. H., Bjelland, M., Lien, N., Andersen, L. F., Ommundsen, Y., Klepp, K. I., & Anderssen, S. A. (2013). Intervention effects on physical activity: the HEIA study - a cluster randomized controlled trial. *The International Journal of Behavioral Nutrition and Physical Activity, 10*, 17. doi:10.1186/1479-5868-10-17

Guthrie, N., Bradlyn, A., Thompson, S. K., Yen, S., Haritatos, J., Dillon, F., & Cole, S. W. (2015). Development of an accelerometer-linked online intervention system to promote physical activity in adolescents. *10*(5), e0128639. doi:10.1371/journal.pone.0128639

Haerens, L., De Bourdeaudhuij, I., Maes, L., Cardon, G., & Deforche, B. (2007). School-based randomized controlled trial of a physical activity intervention among adolescents. *Journal of Adolescent Health, 40*(3), 258-265.

Haerens, L., Deforche, B., Vandelanotte, C., Maes, L., & De Bourdeaudhuij, B. (2007). Acceptability, feasibility and effectiveness of a computer-tailored physical activity intervention in adolescents. *Patient Education and Counseling, 66*. doi:10.1016/j.pec.2007.01.003

Hardman, C. A., Horne, P. J., & Lowe, C. F. (2009). A Home-Based Intervention to Increase Physical Activity in Girls: The Fit ‘n’ Fun Dudes Program. *Journal of Exercise Science & Fitness, 7*(1), 1-8. doi:10.1016/S1728-869X(09)60001-0

Hovell, M. F., Nichols, J. F., Irvin, V. L., Schmitz, K. E., Rock, C. L., Hofstetter, C. R., Keating, K., & Stark, L. J. (2009). Parent/Child training to increase preteens' calcium, physical activity, and bone density: a controlled trial. *American Journal of Health Promotion, 24*(2), 118-128. doi:10.4278/ajhp.08021111

Jago, R., McMurray, R. G., Drews, K. L., Moe, E. L., Murray, T., Pham, T. H., Venditti, E. M., & Volpe, S. L. (2011). HEALTHY intervention: fitness, physical activity, and metabolic syndrome results. *Medicine and Science in Sports and Exercise, 43*(8), 1513-1522. doi:10.1249/MSS.0b013e31820c9797

Jago, R., Sebire, S. J., Davies, B., Wood, L., Edwards, M. J., Banfield, K., Fox, K. R., Thompson, J. L., Powell, J. E., & Montgomery, A. A. (2014). Randomised feasibility trial of a teaching assistant led extracurricular physical activity intervention for 9 to 11 year olds: Action 3:30. *International Journal of Behavioral Nutrition and Physical Activity, 11*, 114. doi:10.1186/s12966-014-0114-z

Jemmott, J. B., 3rd, Jemmott, L. S., O'Leary, A., Ngwane, Z., Icard, L., Bellamy, S., Jones, S., Landis, J. R., Heeren, G. A., Tyler, J. C., & Makiwane, M. B. (2011). Cognitive-behavioural health-promotion intervention increases fruit and vegetable consumption and physical activity among South African adolescents: a cluster-randomised controlled trial. *Psychology & Health, 26*(2), 167-185. doi:10.1080/08870446.2011.531573

Klesges, R. C., Obarzanek, E., Kumanyika, S., Murray, D. M., Klesges, L. M., Relyea, G. E., Stockton, M. B., Lanctot, J. Q., Beech, B. M., McClanahan, B. S., Sherrill-Mittleman, D., & Slawson, D. L. (2010). The Memphis Girls' health Enrichment Multi-site Studies (GEMS): an evaluation of the efficacy of a 2-year obesity prevention program in African American girls. *Archives of Pediatrics & Adolescent Medicine, 164*(11), 1007-1014. doi:10.1001/archpediatrics.2010.196

Kobel, S., Wirt, T., Schreiber, A., Kesztyus, D., Kettner, S., Erkelenz, N., Wartha, O., & Steinacker, J. M. (2014). Intervention effects of a school-based health promotion programme on obesity related behavioural outcomes. *Journal of Obesity, 2014*, 476230. doi:10.1155/2014/476230

Lanckriet, S., Brissieux, E., Borys, J.-M., Jaruga, A., Schnebelen-Berthier, C., Dekneudt, E., Calais, A., Richard, P., Mayer, J., Bournez, C., Penin, J., Djouak, A., Chieh, A., Normand, A., Duclos, M., & Lecerf, J.-M. (2017). Assessment of the Impact of a Sports Kit on Physical Activity in Children 8 to 11 Years of Age. *Journal of Physical Activity Research, 2*(1), 50-60. doi:10.12691/jpar-2-1-9

Laukkanen, A., Pesola, A. J., Heikkinen, R., Sääkslahti, A. K., & Finni, T. (2015). Family-based cluster randomized controlled trial enhancing physical activity and motor competence in 4–7-year-old children. *PLOS ONE, 10*(10).

Lawlor, D. A., Kipping, R. R., Anderson, E. L., Howe, L. D., Chittleborough, C. R., Moure-Fernandez, A., Noble, S. M., Rawlins, E., Wells, S. L., Peters, T. J., Jago, R., & Campell, R. (2016). *NIHR Journals Library. Public Health Research*, 06. doi:10.3310/phr04070

López-Fernández, I., Molina-Jodar, M., Garrido-González, F. J., Pascual-Martos, C. A., Chinchilla Minguet, J. L., & Carnero, E. A. (2016). Promoting physical activity at the school playground: a quasi-experimental intervention study. *Journal of Human Sport and Exercise, 11*(2). doi:10.14198/jhse.2016.112.05

Lubans, D., Morgan, P., Okely, A., Dewar, D., Collins, C., Batterham, M., Callister, R., & Plotnikoff, R. (2012). Preventing obesity among adolescent girls: Outcomes of the nutrition and enjoyable activity for teen girls cluster randomized controlled trial. *Journal of Science and Medicine in Sport, 15 (SUPPL.1)*, S332.

Lubans, D. R., Morgan, P. J., Aguiar, E. J., & Callister, R. (2011). Randomized controlled trial of the physical activity leaders (PALs) program for adolescent boys from disadvantaged secondary schools. *Preventive Medicine: An International Journal Devoted to Practice and Theory, 52*(3-4), 239-246.

Lubans, D. R., Smith, J. J., Plotnikoff, R. C., Dally, K. A., Okely, A. D., Salmon, J., & Morgan, P. J. (2016). Assessing the sustained impact of a school-based obesity prevention program for adolescent boys: the ATLAS cluster randomized controlled trial. *International Journal of Behavioral Nutrition and Physical Activity, 13*, 92. doi:10.1186/s12966-016-0420-8

Manios, Y., Kafatos, I., & Kafatos, A. (2006). Ten-year follow-up of the Cretan Health and Nutrition Education Program on children's physical activity levels. *Preventive Medicine: An International Journal Devoted to Practice and Theory, 43*(6), 442-446. doi:10.1016/j.ypmed.2006.06.001

McNeil, D. A., Wilson, B. N., Siever, J. E., Ronca, M., & Mah, J. K. (2009). Connecting children to recreational activities: results of a cluster randomized trial. *American Journal of Health Promotion, 23*(6), 376-387. doi:10.4278/ajhp.071010107

Mehtälä, M. A., Sääkslahti, A., Soini, A., Tammelin, T., Kulmala, J., Villberg, J., Nissinen, K., & Poskiparta, M. (2018). The effect of the cluster randomized HIPPA intervention on childcare children's overall physical activity. *Baltic Journal of Health & Physical Activity, 9*(4), 89-111.

Meier, M. D., Hager, R. L., Vincent, S. D., Tucker, L. A., & Vincent, W. J. (2007). The Effects of Leisure-Based Screen Time. *American Journal of Health Education, 38*(3), 139-146.

Meyer, U., Schindler, C., Zahner, L., Ernst, D., Hebestreit, H., van Mechelen, W., Brunner-La Rocca, H. P., Probst-Hensch, N., Puder, J. J., & Kriemler, S. (2014). Long-term effect of a school-based physical activity program (KISS) on fitness and adiposity in children: a cluster-randomized controlled trial. *PLOS ONE, 9*(2). doi:10.1371/journal.pone.0087929

Morgan, P. J., Lubans, D. R., Callister, R., Okely, A. D., Burrows, T. L., Fletcher, R., & Collins, C. E. (2011). The 'Healthy Dads, Healthy Kids' randomized controlled trial: efficacy of a healthy lifestyle program for overweight fathers and their children. *International Journal of Obesity, 35*(3), 436-447. doi:10.1038/ijo.2010.151

Morgan, P. J., Young, M. D., Barnes, A. T., Eather, N., Pollock, E. R., & Lubans, D. R. (2018). Engaging Fathers to Increase Physical Activity in Girls: The "Dads And Daughters Exercising and Empowered" (DADEE) Randomized Controlled Trial. *Annals of Behavioral Medicine*, 10. doi:10.1093/abm/kay015

Morris, J. G., Gorely, T., Sedgwick, M. J., Nevill, A., & Nevill, M. E. (2013). Effect of the Great Activity Programme on healthy lifestyle behaviours in 7-11 year olds. *Journal of Sports Sciences, 31*(12), 1280-1293. doi:10.1080/02640414.2013.781665

Murillo Pardo, B., Garcia Bengoechea, E., Julian Clemente, J. A., & Generelo Lanaspa, E. (2016). Motivational Outcomes and Predictors of Moderate-to-Vigorous Physical Activity and Sedentary Time for Adolescents in the Sigue La Huella Intervention. *International Journal of Behavioral Medicine, 23*(2), 135-142. doi:10.1007/s12529-015-9528-5

Ni Mhurchu, C., Maddison, R., Jiang, Y., Jull, A., Prapavessis, H., & Rodgers, A. (2008). Couch potatoes to jumping beans: a pilot study of the effect of active video games on physical activity in children. *International Journal of Behavioral Nutrition and Physical Activity, 5*, 8. doi:10.1186/1479-5868-5-8

Nyberg, G., Norman, A., Sundblom, E., Zeebari, Z., & Elinder, L. S. (2016). Effectiveness of a universal parental support programme to promote health behaviours and prevent overweight and obesity in 6-year-old children in disadvantaged areas, the Healthy School Start Study II, a cluster-randomised controlled trial. *International Journal of Behavioral Nutrition and Physical Activity, 13*, 4. doi:10.1186/s12966-016-0327-4

Nyberg, G., Sundblom, E., Norman, A., Bohman, B., Hagberg, J., & Elinder, L. S. (2015). Effectiveness of a universal parental support programme to promote healthy dietary habits and physical activity and to prevent overweight and obesity in 6-year-old children: the Healthy School Start Study, a cluster-randomised controlled trial. *PLOS ONE, 10*(2). doi:10.1371/journal.pone.0116876

O'Dwyer, M. V., Fairclough, S. J., Knowles, Z., & Stratton, G. (2012). Effect of a family focused active play intervention on sedentary time and physical activity in preschool children. *International Journal of Behavioral Nutrition and Physical Activity, 9*, 117. doi:10.1186/1479-5868-9-117

Okely, A. D., Lubans, D. R., Morgan, P. J., Cotton, W., Peralta, L., Miller, J., Batterham, M., & Janssen, X. (2017). Promoting physical activity among adolescent girls: the Girls in Sport group randomized trial. *International Journal of Behavioral Nutrition and Physical Activity, 14*(1), 81. doi:10.1186/s12966-017-0535-6

Pardo, B. M., Bengoechea, E. G., Julian Clemente, J. A., & Lanaspa, E. G. (2014). Empowering adolescents to be physically active: three-year results of the Sigue la Huella intervention. *Preventive Medicine, 66*, 6-11. doi:10.1016/j.ypmed.2014.04.023

Pate, R. R., Ward, D. S., Saunders, R. P., Felton, G., Dishman, R. K., & Dowda, M. (2005). Promotion of physical activity among high-school girls: a randomized controlled trial. *American Journal of Public Health, 95*(9), 1582-1587.

Patrick, K., Calfas, K. J., Norman, G. J., Zabinski, M. F., Sallis, J. F., Rupp, J., Covin, J., & Cella, J. (2006). Randomized controlled trial of a primary care and home-based intervention for physical activity and nutrition behaviors: PACE+ for adolescents. *Archives of Pediatrics & Adolescent Medicine, 160*(2), 128-136.

Penalvo, J. L., Sotos-Prieto, M., Santos-Beneit, G., Pocock, S., Redondo, J., & Fuster, V. (2013). The Program SI! intervention for enhancing a healthy lifestyle in preschoolers: first results from a cluster randomized trial. *BMC Public Health, 13*, 1208. doi:10.1186/1471-2458-13-1208

Pienaar, A. E., Salome Kruger, H., Steyn, H. S., & Naude, D. (2012). Change over three years in adolescents' physical activity levels and patterns after a physical activity intervention: play study. *Journal of Sports Medicine and Physical Fitness, 52*(3), 300-310.

Razak, L. A., Yoong, S. L., Wiggers, J., Morgan, P. J., Jones, J., Finch, M., Sutherland, R., Lecathelnais, C., Gillham, K., Clinton-McHarg, T., & Wolfenden, L. (2018). Impact of scheduling multiple outdoor free-play periods in childcare on child moderate-to-vigorous physical activity: a cluster randomised trial. *International Journal of Behavioral Nutrition and Physical Activity, 15*(1), 34. doi:10.1186/s12966-018-0665-5

Reilly, J. J., Kelly, L., Montgomery, C., Williamson, A., Fisher, A., McColl, J. H., Lo Conte, R., Paton, J. Y., & Grant, S. (2006). Physical activity to prevent obesity in young children: cluster randomised controlled trial. *BMJ, 333*(7577), 1041.

Robbins, L. B., Ling, J., Sharma, D. B., Dalimonte-Merckling, D. M., Voskuil, V. R., Resnicow, K., Kaciroti, N., & Pfeiffer, K. A. (2018). Intervention Effects of "Girls on the Move" on Increasing Physical Activity: A Group Randomized Trial. *Annals of Behavioral Medicine*, 07. doi:10.1093/abm/kay054

Robbins, L. B., Pfeiffer, K. A., Maier, K. S., Lo, Y. J., & Wesolek Ladrig, S. M. (2012). Pilot intervention to increase physical activity among sedentary urban middle school girls: a two-group pretest-posttest quasi-experimental design. *Journal of School Nursing, 28*(4), 302-315. doi:10.1177/1059840512438777

Rosenkranz, R. R., Behrens, T. K., & Dzewaltowski, D. A. (2010). A group-randomized controlled trial for health promotion in Girl Scouts: healthier troops in a SNAP (Scouting Nutrition & Activity Program). *BMC Public Health, 10*, 81. doi:10.1186/1471-2458-10-81

Rostami-Moez, M., Rezapur-Shahkolai, F., Hazavehei, S. M., Karami, M., Karimi-Shahanjarini, A., & Nazem, F. (2017). Effect of Educational Program, Based on PRECEDE and Trans-Theoretical Models, on Preventing Decline in Regular Physical Activity and Improving it among Students. *Journal of Research in Health Sciences, 17*(2), e00375.

Salminen, M., Vahlberg, T., Ojanlatva, A., & Kivela, S. L. (2005). Effects of a controlled family-based health education/counseling intervention. *American Journal of Health Behavior, 29*(5), 395-406.

Salmon, J., Ball, K., Hume, C., Booth, M., & Crawford, D. (2008). Outcomes of a group-randomized trial to prevent excess weight gain, reduce screen behaviours and promote physical activity in 10-year-old children: switch-play. *International Journal of Obesity, 32*(4), 601-612. doi:10.1038/sj.ijo.0803805

Salmon, J., Jorna, M., Hume, C., Arundell, L., Chahine, N., Tienstra, M., & Crawford, D. (2010). A translational research intervention to reduce screen behaviours and promote physical activity among children: Switch-2-Activity. *Health promotion international, 26*(3), 311-321. doi:10.1093/heapro/daq078

Sanaeinasab, H., Saffari, M., Pakpour, A. H., Nazeri, M., & Piper, C. N. (2012). A model-based educational intervention to increase physical activity among Iranian adolescents. *Jornal de Pediatria, 88*(5), 430-438. doi:10.2223/JPED.2223

Schneider, M., & Cooper, D. M. (2011). Enjoyment of exercise moderates the impact of a school-based physical activity intervention. *International Journal of Behavioral Nutrition and Physical Activity, 8*, 64. doi:10.1186/1479-5868-8-64

Schneider, M., Dunton, G. F., & Cooper, D. M. (2008). Physical Activity and Physical Self-Concept among Sedentary Adolescent Females; An Intervention Study. *Psychology of Sport & Exercise, 9*(1), 1-14.

Schofield, L., Mummery, W. K., & Schofield, G. (2005). Effects of a controlled pedometer-intervention trial for low-active adolescent girls. *Medicine and Science in Sports and Exercise, 37*(8), 1414-1420.

Sebire, S. J., Jago, R., Banfield, K., Edwards, M. J., Campbell, R., Kipping, R., Blair, P. S., Kadir, B., Garfield, K., Matthews, J., Lyons, R. A., & Hollingworth, W. (2018). Results of a feasibility cluster randomised controlled trial of a peer-led school-based intervention to increase the physical activity of adolescent girls (PLAN-A). *International Journal of Behavioral Nutrition and Physical Activity, 15*(1), 50. doi:10.1186/s12966-018-0682-4

Sigmund, E., El Ansari, W., & Sigmundova, D. (2012). Does school-based physical activity decrease overweight and obesity in children aged 6-9 years? A two-year non-randomized longitudinal intervention study in the Czech Republic. *BMC Public Health, 12*, 570. doi:10.1186/1471-2458-12-570

Smith, J. J., Morgan, P. J., Plotnikoff, R. C., Dally, K. A., Salmon, J., Okely, A. D., Finn, T. L., & Lubans, D. R. (2014). Smart-phone obesity prevention trial for adolescent boys in low-income communities: the ATLAS RCT. *Pediatrics, 134*(3), e723-731. doi:10.1542/peds.2014-1012

Smith, M., Hosking, J., Woodward, A., Witten, K., MacMillan, A., Field, A., Baas, P., & Mackie, H. (2017). Systematic literature review of built environment effects on physical activity and active transport - an update and new findings on health equity. *International Journal of Behavioral Nutrition and Physical Activity, 14*, 27. doi:10.1186/s12966-017-0613-9

Smpokos, E. A., Linardakis, M., Kogevinas, M., & Kafatos, A. G. (2010). Does a health education program for 5-8 year old children improve physical activity and fitness indices? *International Journal of Child Health and Human Development, 3*(1), 57-68.

Stevens, J., Story, M., Ring, K., Murray, D. M., Cornell, C. E., Juhaeri, & Gittelsohn, J. (2003). The impact of the Pathways intervention on psychosocial variables related to diet and physical activity in American Indian schoolchildren. *Preventive Medicine, 37*(6 Pt 2), S70-79.

Story, M., Sherwood, N. E., Himes, J. H., Davis, M., Jacobs, D. R., Jr., Cartwright, Y., Smyth, M., & Rochon, J. (2003). An after-school obesity prevention program for African-American girls: the Minnesota GEMS pilot study. *Ethnicity and Disease, 13*(1 Suppl 1), S54-64.

Sutherland, R., Campbell, E., Lubans, D. R., Morgan, P. J., Okely, A. D., Nathan, N., Wolfenden, L., Wiese, J., Gillham, K., Hollis, J., & Wiggers, J. (2016a). 'Physical Activity 4 Everyone' school-based intervention to prevent decline in adolescent physical activity levels: 12 month (mid-intervention) report on a cluster randomised trial. *British Journal of Sports Medicine, 50*(8), 488-495. doi:10.1136/bjsports-2014-094523

Sutherland, R. L., Campbell, E. M., Lubans, D. R., Morgan, P. J., Nathan, N. K., Wolfenden, L., Okely, A. D., Gillham, K. E., Hollis, J. L., Oldmeadow, C. J., Williams, A. J., Davies, L. J., Wiese, J. S., Bisquera, A., & Wiggers, J. H. (2016b). The Physical Activity 4 Everyone Cluster Randomized Trial: 2-Year Outcomes of a School Physical Activity Intervention Among Adolescents. *American Journal of Preventive Medicine, 51*(2), 195-205. doi:10.1016/j.amepre.2016.02.020

Tarp, J., Domazet, S. L., Froberg, K., Hillman, C. H., Andersen, L. B., & Bugge, A. (2016). Effectiveness of a School-Based Physical Activity Intervention on Cognitive Performance in Danish Adolescents: LCoMotion-Learning, Cognition and Motion - A Cluster Randomized Controlled Trial. *11*(6), e0158087. doi:10.1371/journal.pone.0158087

Taymoori, P., Niknami, S., Berry, T., Lubans, D., Ghofranipour, F., & Kazemnejad, A. (2008). A school-based randomized controlled trial to improve physical activity among Iranian high school girls. *International Journal of Behavioral Nutrition and Physical Activity, 5*, 18. doi:10.1186/1479-5868-5-18

Telford, R. M., Olive, L. S., Cochrane, T., Davey, R., & Telford, R. D. (2016). Outcomes of a four-year specialist-taught physical education program on physical activity: a cluster randomized controlled trial, the LOOK study. *International Journal of Behavioral Nutrition and Physical Activity, 13*, 64. doi:10.1186/s12966-016-0388-4

Toftager, M., Christiansen, L. B., Ersboll, A. K., Kristensen, P. L., Due, P., & Troelsen, J. (2014). Intervention effects on adolescent physical activity in the multicomponent SPACE study: a cluster randomized controlled trial. *PLOS ONE, 9*(6). doi:10.1371/journal.pone.0099369

Vasickova, J., Groffik, D., Fromel, K., Chmelik, F., & Wasowicz, W. (2013). Determining gender differences in adolescent physical activity levels using IPAQ long form and pedometers. *Annals of Agricultural and Environmental Medicine, 20*(4), 749-755.

Verbestel, V., De Henauw, S., Barba, G., Eiben, G., Gallois, K., Hadjigeorgiou, C., Konstabel, K., Maes, L., Marild, S., Molnar, D., Moreno, L. A., Oja, L., Pitsiladis, Y., Ahrens, W., Pigeot, I., De Bourdeaudhuij, I., & consortium, I. (2015). Effectiveness of the IDEFICS intervention on objectively measured physical activity and sedentary time in European children. *Obesity Reviews, 16 Suppl 2*, 57-67. doi:10.1111/obr.12348

Verloigne, M., Bere, E., Van Lippevelde, W., Maes, L., Lien, N., Vik, F. N., Brug, J., Cardon, G., & De Bourdeaudhuij, I. (2012). The effect of the UP4FUN pilot intervention on objectively measured sedentary time and physical activity in 10-12 year old children in Belgium: the ENERGY-project. *BMC Public Health, 12*, 805. doi:10.1186/1471-2458-12-805

Verstraete, S. J., Cardon, G. M., De Clercq, D. L., & De Bourdeaudhuij, I. M. (2007). A comprehensive physical activity promotion programme at elementary school: the effects on physical activity, physical fitness and psychosocial correlates of physical activity. *Public Health Nutrition, 10*(5), 477-484.

Wang, Z., Xu, F., Ye, Q., Tse, L. A., Xue, H., Tan, Z., Leslie, E., Owen, N., & Wang, Y. (2018). Childhood obesity prevention through a community-based cluster randomized controlled physical activity intervention among schools in china: the health legacy project of the 2nd world summer youth olympic Games (YOG-Obesity study). *International Journal of Obesity, 42*(4), 625-633. doi:10.1038/ijo.2017.243

Weaver, R. G., Brazendale, K., Chandler, J. L., Turner-McGrievy, G. M., Moore, J. B., Huberty, J. L., Ward, D. S., & Beets, M. W. (2017). First year physical activity findings from turn up the HEAT (Healthy Eating and Activity Time) in summer day camps. *12*(3), e0173791. doi:10.1371/journal.pone.0173791
